# Molecular identification and phenotypic study of a novel *HBB*: c.-23A>G mutation in the 5’ untranslated region

**DOI:** 10.3389/fmed.2025.1675600

**Published:** 2025-10-30

**Authors:** Shichun Shen, Jungao Huang, Haimei Qi, Zezhang Liu, Wenqian Zhang, Xianping Yuan, Zhuling Zhang, Haijun Chen, Xinxing Xie, Lin Xiao, Junkun Chen, Liyun Song

**Affiliations:** ^1^Department of Medical Genetics, Ganzhou Maternal and Child Health Hospital, Ganzhou, China; ^2^Department of Clinical Laboratory, Ganzhou Maternal and Child Health Hospital, Ganzhou, China; ^3^BGI Genomics, Shenzhen, China; ^4^Clin Lab, BGI Genomics, Wuhan, China; ^5^Obstetrical Department, Ganzhou Maternal and Child Health Hospital, Ganzhou, China; ^6^Department of Clinical Laboratory, Ganzhou People’s Hospital, Ganzhou, China

**Keywords:** β-globin gene, 5′ untranslated region, novel mutation, hematological parameters, minimum free energy

## Abstract

**Background:**

β-thalassemia is a prevalent genetic disorder in the Gannan region, Southern China. Mutations in the 5′ untranslated region of the β-globin gene are associated with diverse clinical phenotypes, posing challenges for effective prevention strategies in this region.

**Methods:**

In this study, carriers of the *HBB*: c.-23A>G mutation were identified from a cohort of 192,720 individuals who underwent thalassemia gene testing in the Gannan region. Hematological data from these carriers were collected, and pedigree information was gathered for further analysis.

**Results:**

Among the 192,720 individuals tested, 75 carriers of the *HBB*: c.-23A>G mutation were identified, yielding a carrier frequency of 3.89 per 10,000. Statistical analysis showed no significant differences in hematological parameters between *HBB*: c.-23A>G heterozygotes and normal individuals. Furthermore, the minimum free energy of mRNA with the *HBB*: c.-23A>G mutation showed no significant difference compared to that of the wild-type mRNA.

**Conclusion:**

The carrier frequency of *HBB*: c.-23A>G in the Gannan region is non-negligible. Hematological data analyses suggested that this mutation may be a likely benign variant. Overall, this study elucidates the molecular and phenotypic characteristics of the *HBB*: c.-23A>G mutation, providing crucial evidence for genetic counseling in clinical practice.

## 1 Introduction

Thalassemia is a group of autosomal recessive genetic disorders caused by reduced or absent synthesis of the globin chains that constitute the hemoglobin (Hb) tetramer (1). Based on the type of globin chain affected, thalassemia is primarily classified into two categories: *α*-thalassemia and β-thalassemia. β-thalassemia is prevalent in southern China, with a carrier frequency ranging from 2.27 to 6.43% (2–4). Gannan, a region in southern China with a population of approximately 8.99 million, has previously reported a β-thalassemia carrier rate of 4.06% (5). Patients with severe β-thalassemia (β-thalassemia major, β-TM) require regular blood transfusions or hematopoietic stem cell transplantation to survive, posing significant medical and public health challenges worldwide, particularly in Southeast Asia and southern China (6).

The transcriptional initiation of human β-globin mRNA occurs at the canonical Cap site (+1) located within the gene’s promoter region (7). Between this Cap site and the translation initiation codon (ATG) lies a 50-nucleotide 5′ untranslated region (UTR) that serves critical regulatory functions in mRNA stability, ribosomal scanning efficiency, and translational control (8, 9). For example, well-characterized mutations in the Kozak sequence within this region have been established as a significant molecular etiology of β-thalassemia (10). Although this region was historically considered to have low mutational susceptibility, the widespread implementation of next-generation sequencing (NGS) in thalassemia carrier screening has revealed an expanding spectrum of pathogenic variants within this regulatory domain, such as *HBB*: c.-40C>G, *HBB*: c.-10A>T, *HBB*: c.-8C>G, and *HBB*: c.-29G>T (11, 12). These newly identified mutations exhibit remarkable phenotypic variability, ranging from silent carrier states to severe transfusion-dependent anemia, thereby complicating genetic counseling and posing challenges to existing public health strategies for thalassemia prevention in endemic regions (13, 14).

In this study, we investigated the clinical phenotype of the *HBB*: c.-23A>G mutation (CAP+28 [A>G]) located in the 5′ UTR of the β-globin gene. Notably, this mutation has not been reported in the specialized globin gene variant databases HbVar^1^ and IthaGenes^2^. Through NGS, we identified 75 carriers of this mutation among 192,720 individuals. We studied the hematological data of these carriers to determine the clinical phenotype of this variant. Our study provides a theoretical basis for the screening, prevention, and treatment of thalassemia in this region and other areas.

## 2 Materials and methods

### 2.1 Participant recruitment

This study included a total of 192,720 participants, covering thalassemia gene testing data from two groups. The first group consisted of 136,312 individuals of childbearing age who were either registered residents of Gannan or lived in Gannan on a regular basis, and the data were collected from April 2019 to April 2021. The second group included 56,408 pregnant women who were either registered residents of Gannan or lived in Gannan on a regular basis, with data collected from April 2023 to December 2024. In addition, the study integrated hematological data from 75 individuals carrying the *HBB*: c.-23A>G mutation, as well as relevant information from 22 pedigrees. All participants signed written informed consent forms before the study began. The study was approved by the Ethics Committee of Ganzhou Maternal and Child Health Hospital (Approval Number: 2024108) and was conducted in accordance with the ethical guidelines for research involving human subjects.

### 2.2 Sample collection and genomic DNA extraction

First, 2 mL of peripheral blood samples were collected using ethylene diamine tetraacetic acid K2 (EDTA-K2) anticoagulated tubes. Genomic DNA was extracted from 200 μL whole blood samples using the QIAamp DNA blood Mini kit (Qiagen, Hilden, Germany). DNA extracts were then arrayed in 96-well plates, and concentration was quantified using a qubit 3.0 fluorometer (Thermo fisher scientific, Waltham, MA, USA). All samples were required to have a DNA concentration >10 ng/μL and an A260/A280 ratio between 1.8 and 2.0 for subsequent analysis.

### 2.3 Next-generation sequencing

The polymerase chain reaction (PCR) technique was employed to amplify the *HBA1*, *HBA2*, and *HBB* genes. The *HBA1* and *HBA2* genes were amplified from 35 bp upstream of the cap site to 150 bp downstream of the termination codon, generating amplicons of approximately 900 bp. Amplification of the *HBB* gene was divided into two segments. The first segment extended from 135 bp upstream of the CAP site at the 5’end to 150 bp into the second intron, generating a DNA fragment of approximately 760 bp. The second segment spanned from 630 bp into the second intron to 150 bp downstream of the termination codon at the 3’end, producing a DNA fragment of approximately 560 bp. After amplification, the DNA fragments were sheared into approximately 200 bp segments by ultrasonication. Following end-repair, sequencing adapters were ligated to construct the sequencing library. Sequencing was carried out on the MGISEQ-200 chip (MGI, Shenzhen, China) in paired-end (PE100) mode. Finally, sequencing data were analyzed using the Halos system to identify mutation sites in the relevant genes.

### 2.4 Site verification and pedigree verification

For positive samples carrying the *HBB*: c.-23A>G mutation, the variant was verified by Sanger sequencing (ABI 310, Applied Biosystems, USA). To exclude other potential mutations in the *HBB* gene, DNA samples were also subjected to third-generation sequencing (Guangdong Hybribio Biotechnology Co., Ltd., Guangzhou, China). Additionally, 22 family members were tested to determine the cis/trans configuration of two compound heterozygotes and to confirm co-segregation of the *HBB*: c.-23A>G mutation in the pedigrees.

### 2.5 Hematological data and analysis

Hematological parameters were measured using the Mindray 7,500 blood cell analyzer (Shenzhen, China). Hemoglobin electrophoresis analysis was performed with the Capillarys 3 capillary electrophoresis instrument (Sebia, France). Hematological data were collected from 80 healthy adults (40 males and 40 females), 60 β^0^ heterozygote carriers (30 males and 30 females), 30 β^+^ heterozygote carriers (15 males and 15 females), 30 β^N^/β^N^ with --^SEA^/αα carriers (15 males and 15 females), 20 β^0^/β^N^ with --^SEA^/αα carriers, 10 β^+^/β^N^ with --^SEA^/αα carriers, and 20 *HBB*: c.-11_-8delAACA and 10 *HBB*: c.-29G>A (Common mutations of 5′ UTR in Gannan region) heterozygotes (10 males and 10 females for the former, 5 males and 5 females for the latter) as the control group (Supplementary Table S1). These data were used to analyze the hematological characteristics of *HBB*: c.-23A>G carriers and explore the potential impact of this mutation on β-chain expression.

### 2.6 Prediction of mRNA secondary structure

Using the RNAfold web server (15), we predicted the secondary structures of *HBB* gene mRNA for the wild type, *HBB*: c.-23A>G mutant, and *HBB*: c.-29G>A mutant. A comparative analysis of the minimum free energy and differences in internal stem-loop structures among the three types of mRNA secondary structures was carried out.

### 2.7 Data analysis and statistics

Statistical analysis was conducted using SPSS 27.0 software. Measured data were expressed as mean ± standard deviation (SD), and count data were expressed as percentage (%). Differences between groups were examined using the independent samples *t*-test. A *p* value of < 0.05 was considered statistically significant.

## 3 Results

### 3.1 Gene testing results of β-thalassemia

After analyzing the screening results of 192,720 individuals, a total of 7,968 β-thalassemia carriers were identified, resulting in a carrier rate of 4.134% in this population. Among these β-thalassemia carriers, 75 individuals were identified as carrying the *HBB*: c.-23A>G mutation, with a carrier frequency of 3.89 per 10,000 in this region. The genotypic distribution was as follows: 65 were β^*HBB*:c.-23A>G^/β^N^ with αα/αα, 7 were β^*HBB*:c.-23A>G^/β^N^ with --^SEA^/αα, 1 was β^*HBB*:c.-23A>G^/β^N^ with -α^3.7^/αα, 1 was β^*HBB*:c.-23A>G^/β^*HBB*:c.126_129delCTTT^ with αα/αα, and 1 was β^*HBB*:c.-23A>G^/β^*HBB*:c.316-197C>T^ with αα/αα. Pedigree follow-up revealed that four carriers had spouses who were also β-thalassemia carriers, and two carriers were sisters (Supplementary Table S2).

### 3.2 Mutation verification

**Figure 1:**
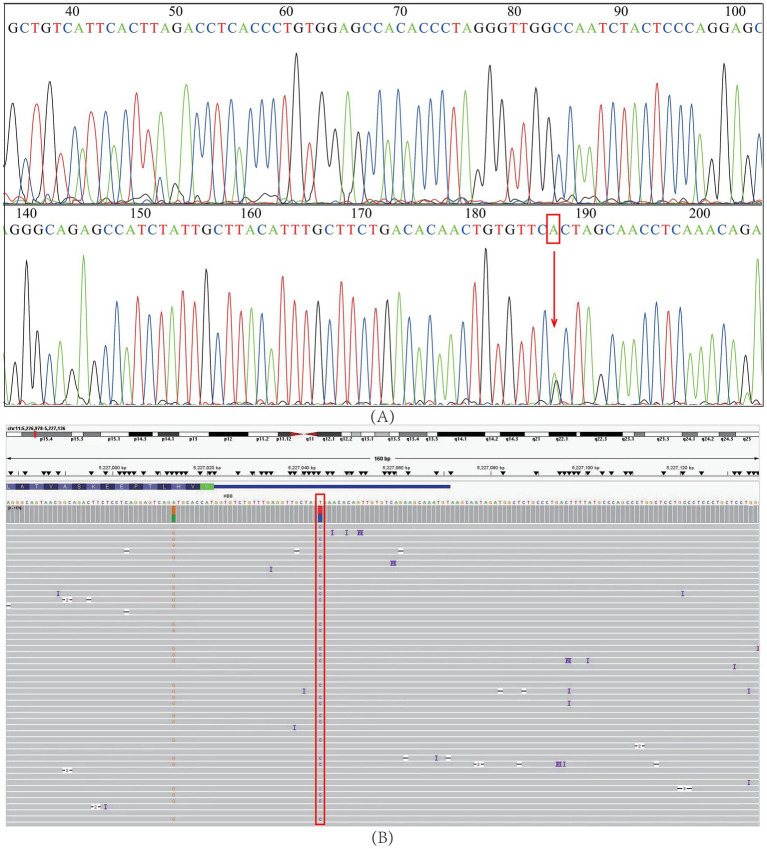
Verification of the HBB: c.-23A>G mutation. **(A)** Sanger sequencing. **(B)** Third-generation sequencing. The red squares and arrow represent the positions of the *HBB*: c.-23A>G mutation.

Sanger sequencing of DNA samples harboring the *HBB*: c.-23A>G mutation validated all NGS results, confirming the accuracy of the NGS method (Figure 1A). Moreover, third-generation sequencing provided additional verification and confirmed the absence of other pathogenic *HBB* gene mutations (Figure 1B).

**Figure 2:**
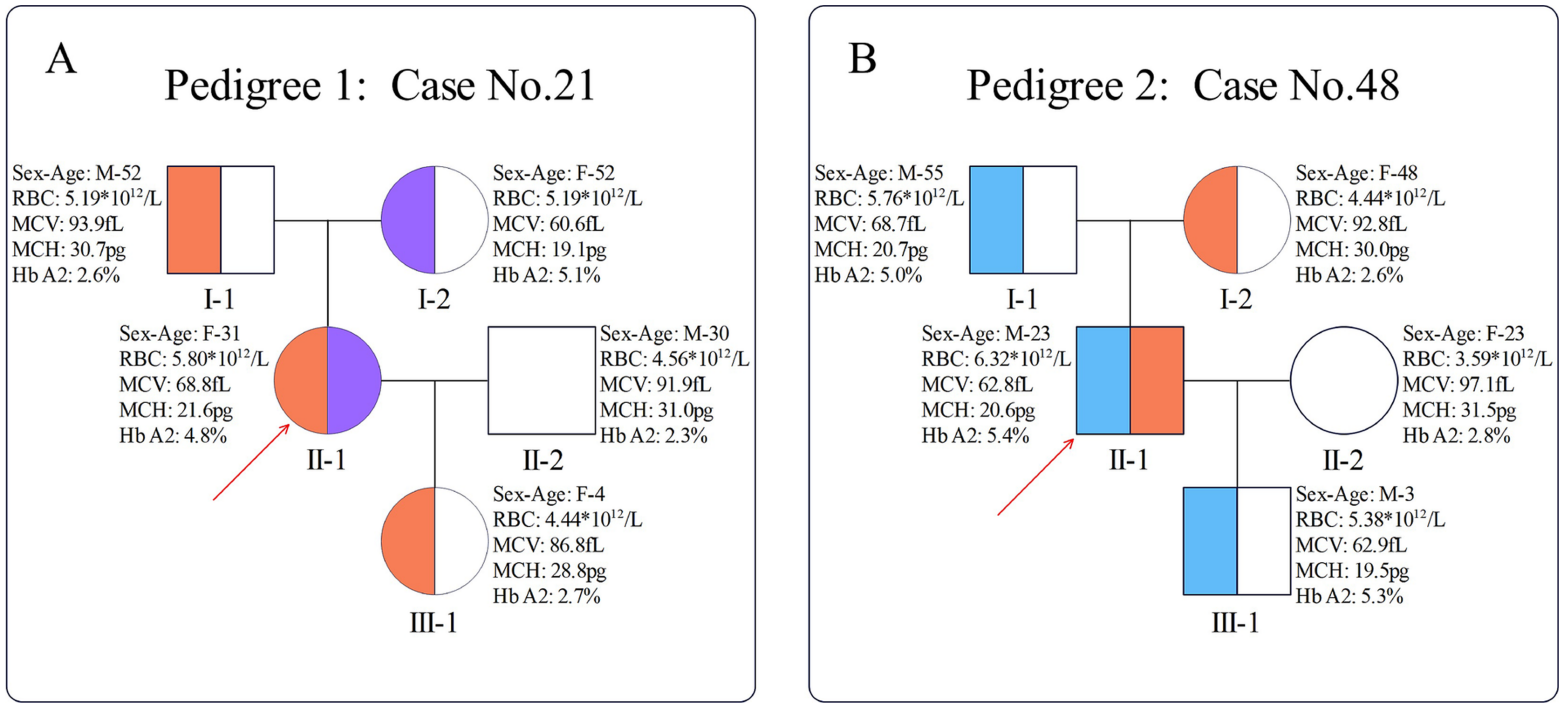
Pedigrees of two compound heterozygotes. **(A)** Pedigree of case 21; **(B)** pedigree of case 48. The red arrow indicates the proband; orange indicates the presence of the *HBB*: c.-23AG mutation, purple indicates the presence of the *HBB*: c.316-197C > T mutation, and blue indicates the presence of the *HBB*: c.126_129delCTTT mutation.

For the carrier with β^*HBB*: c.-23A>G^/β^*HBB*: c.316-197C>T^ genotype, pedigree verification revealed that *HBB*: c.-23A>G was inherited from the father, while *HBB*: c.316-197C>T was inherited from the mother. Additionally, her daughter inherited the *HBB*: c.-23A>G mutation (Figure 2A). In another carrier with β^*HBB*: c.-23A>G^/β^*HBB*: c.126_129delCTTT^ genotype, pedigree verification showed that *HBB*: c.-23A>G was inherited from the mother, while *HBB*: c.126_129delCTTT was inherited from the father. In addition, his son inherited the *HBB*: c.126_129delCTTT mutation (Figure 2B). Notably, among the 18 families with heterozygous carriers, the mutation was transmitted to the next generation in 8 cases (Supplementary Table S3).

### 3.3 Hematological analysis

After excluding the carriers with iron-deficiency anemia, we obtained the hematological data of 69 heterozygotes, 6 heterozygotes with αα/--^SEA^, 1 heterozygote with αα/-α^3.7^, and 2 compound heterozygotes (Supplementary Table S4). The mean corpuscular volume (MCV) of the 69 heterozygotes was 92.07 ± 3.94 fL, while the mean corpuscular hemoglobin (MCH) was 30.58 ± 1.49 pg, and the mean hemoglobin A2 (Hb A2) was 2.70 ± 0.17%. In comparison, the MCV, MCH, and Hb A2 of the 6 heterozygotes with αα/--^SEA^, were 70.07 ± 1.99 fL, 21.58 ± 0.67 pg, and 2.28 ± 0.07%, respectively (Table 1).

**Table 1:** Hematological data statistics of HBB: c.-23A>G mutation carriers.

Genotype	Number of cases	RBC (10^12^/L)	HB (g/L)	MCV (fL)	MCH (pg)	Hb A2 (%)
αα/αα β^*HBB*: c.-23A>G^/β^N^	69	4.45 ± 0.65	135.51 ± 17.84	92.07 ± 3.94	30.58 ± 1.49	2.70 ± 0.17
	25 (Male)	5.07 ± 0.53	153.44 ± 13.97	91.21 ± 3.40	30.30 ± 1.14	2.66 ± 0.17
	44 (Female)	4.09 ± 0.40	125.32 ± 10.10	92.57 ± 4.14	30.73 ± 1.64	2.72 ± 0.17
αα/--^SEA^ β^*HBB*: c.-23A>G^/β^N^	6	5.60 ± 0.33	121.00 ± 9.42	70.07 ± 1.99	21.58 ± 0.67	2.28 ± 0.07
	2 (Male)	5.36,6.25	110,140	6.95,70.1	20.4,22.4	2.2,2.3
	4 (Female)	5.50 ± 0.18	119 ± 2.92	70.20 ± 2.42	21.68 ± 0.40	2.30 ± 0.07
αα/-α^3.7^ β^*HBB*: c.-23A>G^/β^N^	1 (Male)	5.88	160	82.6	27.2	2.6
αα/αα β^*HBB*: c.-23A>G^/β^*HBB*: c.316-197C>T^	1 (Female)	5.80	125	68.8	21.6	4.8
αα/αα β^*HBB*: c.-23A>G^/β^*HBB*:c.126_129delCTTT^	1 (Male)	6.32	130	62.8	20.6	5.4

**Figure 3:**
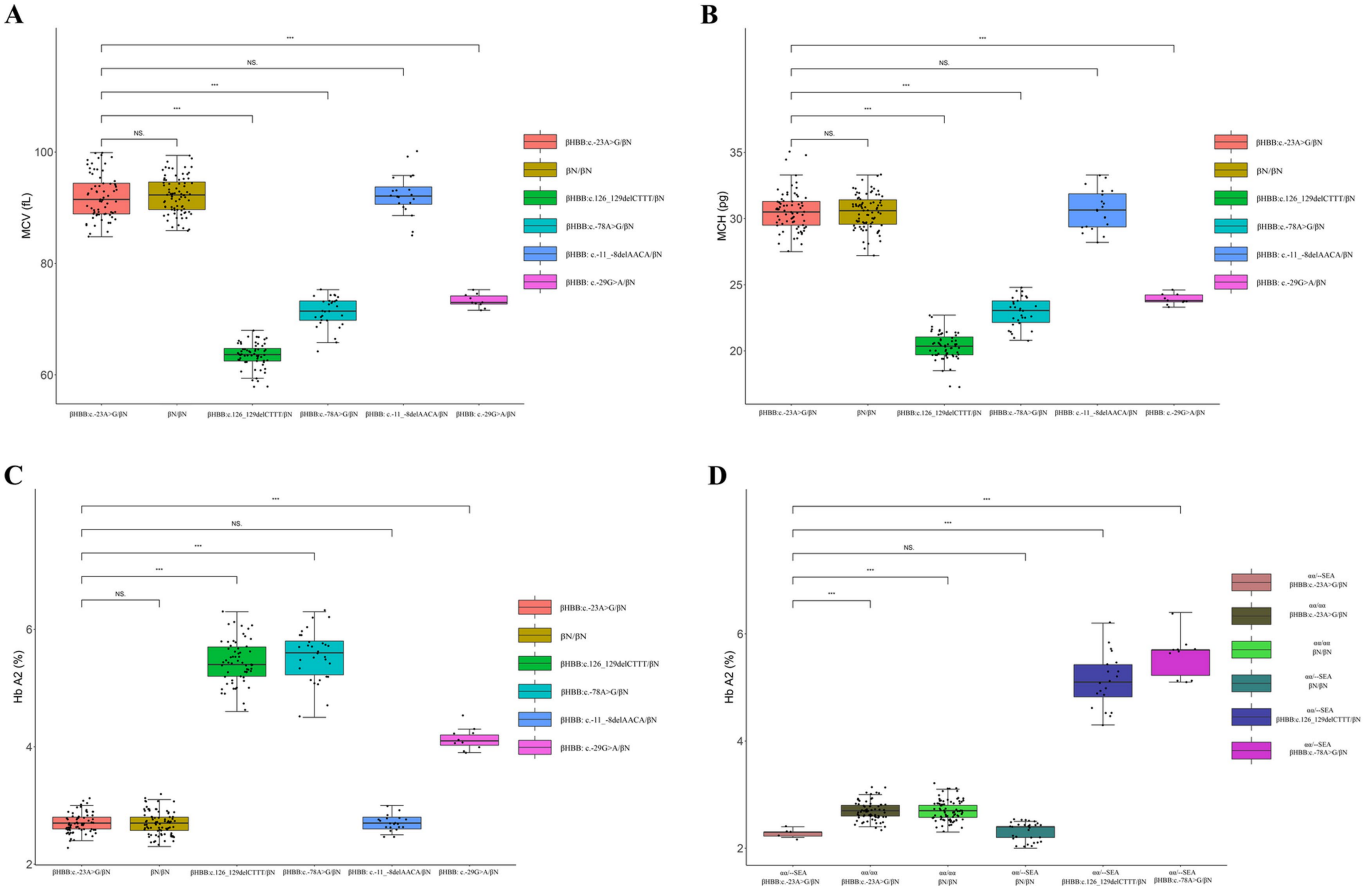
Comparison of hematological data between *HBB* c.-23AG mutation carriers and the control group. **(A)** Comparison of MCV between *HB*B: c.-23A>G heterozygotes and the control group; **(B)** comparison of MCH between *HBB*: c.-23A>G heterozygotes and the control group; **(C)** comparison of Hb A2 between *HBB*: c.-23A>G heterozygotes and the control group; **(D)** comparison of Hb A2 between the β^*HBB*: c.-23A>G^/β^N^ with αα/αα group and the control group. “NS” indicates no statistically significant difference between the two groups, while “***” signifies an extremely significant statistical difference between the two groups (*p* < 0.001).

After comparing with hematological data of control group, the MCV, MCH, and Hb A2 of the 69 heterozygotes showed significant differences (*p* < 0.001) with those of β^0^ heterozygote carriers, β^+^ heterozygote carriers, and *HBB*: c.-29G>A heterozygotes. In contrast, there were no significant differences in MCV, MCH, and Hb A2 between the 69 heterozygotes and the normal population or *HBB*: c.-11_-8delAACA heterozygotes (Figure 3). There were no statistically significant differences in Hb A2 between the 6 compound αα/--^SEA^ heterozygotes and the β^N^/β^N^ with --^SEA^/αα group. However, significant differences were observed when compared with the normal population, the β^*HBB*: c.-23A>G^/β^N^ with αα/αα group, the β^0^/β^N^ with --^SEA^/αα group, and the β^+^/β^N^ with --^SEA^/αα group (Figure 3D).

The two compound heterozygotes (β^HBB:c.-23A>G^/β^HBB:c.316-197C>T^ and β^HBB:c.-23A>G^/β^HBB:c.126_129delCTTT^) exhibited hemoglobin levels of 125 and 130 g/L, respectively, with microcytic hypochromic erythrocytes (MCV: 68.8 fL and 62.8 fL; MCH: 21.6 pg and 20.6 pg) and elevated Hb A2 (4.8 and 5.4%). No elevation of Hb F was found in either of them (Table 1). Both individuals were asymptomatic and did not require transfusion or chelation therapy. Their clinical presentations were consistent with β-thalassemia trait rather than thalassemia intermedia or major.

### 3.4 Secondary structure prediction of mRNA

**Figure 4:**
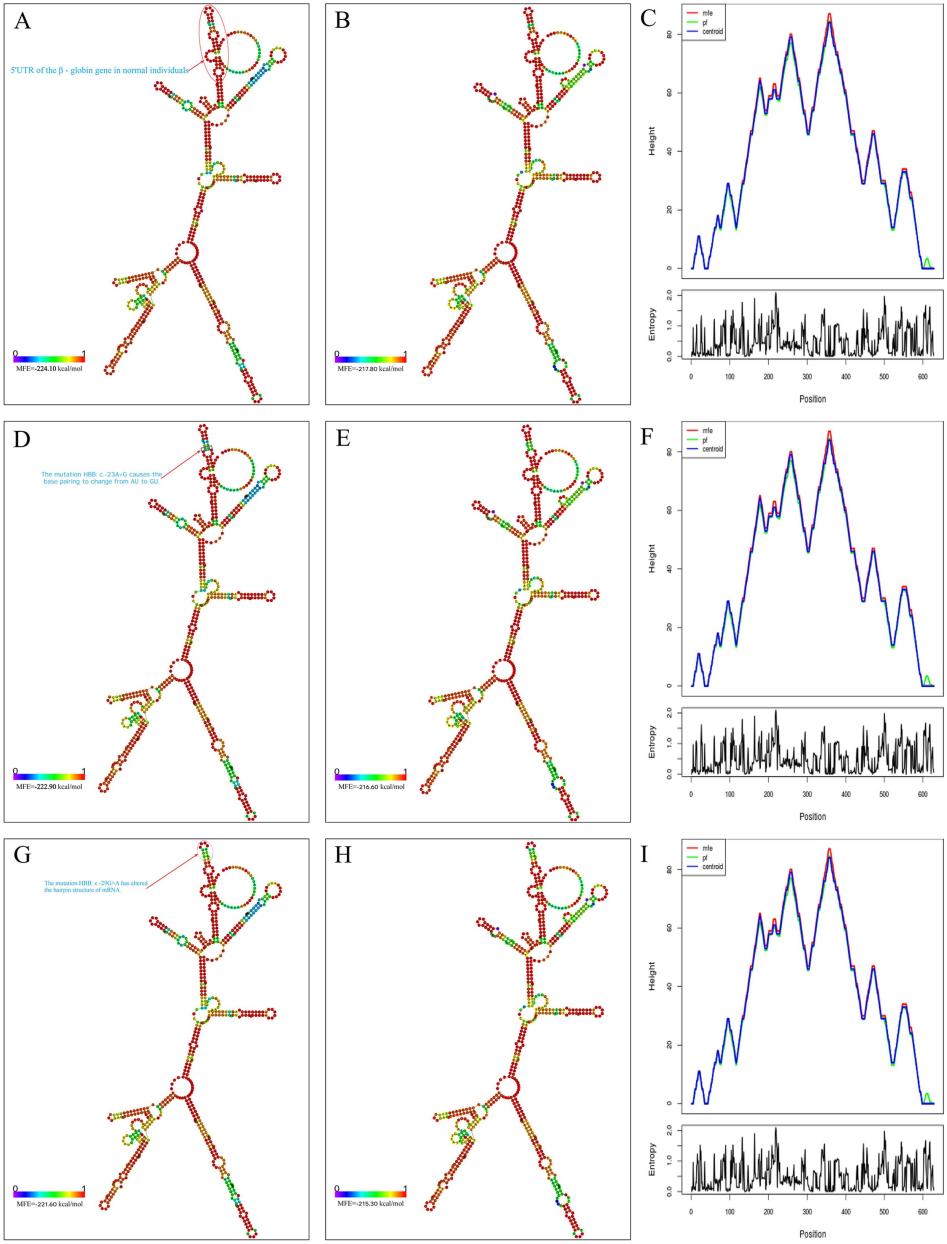
Prediction of mRNA secondary structure. **(A)** MFE structure drawing encoding base-pair probabilities in normal individuals; **(B)** centroid structure drawing encoding base-pair probabilities in normal individuals; **(C)** the binding strength at each site on mRNA in normal individuals; **(D)** MFE structure drawing encoding base-pair probabilities in *HBB*: c.-23A>G mutants; **(E)** centroid structure drawing encoding base-pair probabilities in *HBB*: c.-23A>G mutants; **(F)** the binding strength at each site on mRNA in *HBB*: c.-23A>G mutants; **(G)** MFE structure drawing encoding base-pair probabilities in *HBB*: c.-29G>A mutants; **(H)** centroid structure drawing encoding base-pair probabilities in *HBB*: c.-29G>A mutants; **(I)** the binding strength at each site on mRNA in *HBB*: c.-29G>A mutants.

Secondary structures of wild-type and mutant (*HBB*: c.-23A>G and HBB: c.-29G>A) *HBB* gene mRNA were predicted using the RNAfold web server. We analyzed the minimum free energy (MFE) structures, centroid structures, and per-nucleotide binding probabilities (Figures 4A–I). The MFE values for the wild-type, HBB: c.-23A>G mutant, and HBB: c.-29G>A mutant mRNAs were -224.10 kcal/mol (Figure 4A), -222.90 kcal/mol (Figure 4D), and -221.60 kcal/mol (Figure 4G), respectively. Statistical analysis revealed that the differences among these three MFEs were not statistically significant (*p* > 0.05). Compared with the wild type, the *HBB* gene mRNA of the *HBB*: c.-23A>G mutant exhibited only a change from AU pairing to GU pairing in the first internal loop (Figure 4D). In contrast, the *HBB* gene mRNA of the *HBB*: c.-29G>A mutant altered the structure of the hairpin loop (Figure 4G).

## 4 Discussion

β-thalassemia exhibits a broad global distribution with striking heterogeneity in its mutational spectrum across geographic and ethnic populations. This diversity is evident in the distinct profiles of common pathogenic alleles, such as the IVS-I-110, IVS-I-1, and IVS-I-6 variants prevalent in Turkey and Lebanon (16, 17), which contrast sharply with the IVS-II-654 (*HBB*: c.316-197C>T) and CD41-42 (*HBB*: c.126_129delCTTT) mutations common in China. This principle of geographic stratification extends to the regulatory 5′ UTR, where despite a limited number of reported mutations, distinct ethnogeographic patterns are observed: *HBB*: c.-29G>A is primarily found in Black Sea-bordering countries like Turkey and Bulgaria (18), *HBB*: c.-50A>C has been identified in South Asian Indian populations (19), and *HBB*: c.-11_-8delAACA demonstrates high frequency in China (20).

Functional studies suggest that within this short 50-nucleotide 5′ UTR, different “sub-regions” may be associated with distinct regulatory functions (21). The complex interplay between genotype and phenotype (22), further complicated by population migration spreading thalassemia beyond traditional endemic regions (23), must be considered when evaluating any variant’s clinical significance. Phenotypic variability, even for the same mutation across different reports, often presents diagnostic challenges for clinicians (24). Many studies have attempted to address this challenge by using online prediction tools and sequence interpretation databases, such as the Hb Var database (25), Itha Genes (26), and the LOVD database (27). However, the American College of Medical Genetics and Genomics (ACMG) guidelines explicitly caution against overreliance on in silico pathogenicity predictions, stating they “should not be used as standalone evidence for clinical interpretation” (8). The ACMG framework mandates rigorous evaluation of multiple evidentiary categories, emphasizing that comprehensive evaluation of published clinical data remains imperative when assessing potential variant pathogenicity (28).

Through literature retrieval, we found that the *HBB*: c.-23A>G variant was first submitted to the ClinVar database by Women’s Health and Genetics/Laboratory Corporation of America, LabCorp on March 17, 2018 (Variant ID: rs1010004981) (29). Given the lack of clinical information and functional studies, the variant is classified as a variant of uncertain significance (VUS). In addition, this study identified descriptions of this site in two publications, both of which reported case information of one individual carrying the mutation from China (2, 30). Nonetheless, neither of these two publications conducted in-depth research on this mutation. Based on the information retrieved, it is indeed challenging to determine the pathogenicity of this mutation.

In previous studies, the carrier rate of β-thalassemia in this region was relatively high (5), and the carrier rate in this study is consistent with the previously reported data (4.134% vs. 4.056%). The prevalence of the *HBB*: c.-23A>G variant in this region also represents a non-negligible frequency. Through follow-up, we found that among 75 carriers, four of them had spouses who were also carriers of β-thalassemia. Given that when both husband and wife are carriers of β-thalassemia, there is a risk of having a child with β-TM (31), we performed prenatal diagnosis for one couple when we first identified this variant, due to the unclear phenotype and limitations in genetic counseling capabilities. Fortunately, the diagnosis showed that the fetus is β^*HBB*: c.316-197C>T^/β^N^, highlighting the particular necessity to study the clinical phenotype of *HBB*: c.-23A>G. The literature indicates that globin chain synthesis studies are the established standard for determining the pathogenicity of a variant, as they help to determine whether the variant has led to a reduction in β-globin chains (8). In this study, we performed hemoglobin electrophoresis analysis and hemoglobin component analysis on *HBB*: c.-23A>G carriers in our region to assess the hematological phenotype and indirectly infer whether *HBB*: c.-23A>G truly causes a reduction in β-globin chains.

This study revealed that the MCV, MCH, and Hb A2 levels of heterozygotes with this mutation showed no significant statistical differences compared with those of the normal population, aligning with a previous report (2). Additionally, we observed that the MCV, MCH, and Hb A2 levels of compound heterozygotes carrying this variant and β^0/+^ showed no statistical differences compared with those of β^0/+^ heterozygotes alone. The MCV, MCH, and Hb A2 levels of a previously reported case of β^*HBB*: c.316-197C>T^/β^*HBB*: c.-23A>G^ compound heterozygote also were consistent with the results of this study (30). Crucially, none of these cases exhibited the compensatory high Hb F expression observed in β-TM. The level of Hb A2 is considered an important indicator for assessing the expression of *α*- and β-globin chains (32). When α-thalassemia occurs, the reduced synthesis of α-globin chains leads to a decrease in Hb A2 levels. In contrast, when β-thalassemia occurs, the reduced synthesis of β-globin chains results in an increase in Hb A2 levels. However, in patients who carry both α- and β-thalassemia, despite the reduced synthesis of both α- and β-globin chains, the Hb A2 levels are elevated. We observed that in the heterozygotes for this variant, their Hb A2 levels did not increase. In the cases of β^*HBB* c.-23A>G^/β^N^ with --^SEA^/αα, the Hb A2 levels did not increase but decreased instead. Based on the above data, this study concluded that the *HBB*: c.-23A>G mutation does not lead to a reduction in the production of β-globin chains. This mutation is likely to be mainly distributed in the Chinese population, and its phenotype is consistent with silent β^++^ phenotype. According to the ACMG guidelines, genetic interpretation experts, in conjunction with the data from this study, classified the *HBB*: c.-23A>G mutation as likely benign (BS4 and BP4).

Compared to other known 5′ UTR mutations, *HBB*: c.-23A>G demonstrates a distinct phenotypic profile. This region exhibits a spectrum of phenotypic outcomes across different populations: the *HBB*: c.-29G>A mutation, prevalent in Turkey and Bulgaria, is associated with a typical β^+^ thalassemia phenotype (18); the *HBB*: c.-50A>C mutation, identified in South Asian Indian populations, presents a β^++^ phenotype (19); and the HBB: c.-11_-8delAACA mutation, common in China, manifests as a silent β^++^ phenotype (20). Our study establishes HBB: c.-23A>G as a silent β^++^ variant, based on the absence of significant hematological alterations in heterozygotes. This marked variability underscores the functional diversity within the 5′ UTR and cautions against overgeneralizing the effects of a given mutation across different populations.

The 5′ UTR region downstream of the cap site can be transcribed into mRNA. Mutations in this region primarily reduce β-chain synthesis by affecting mRNA stability and translational efficiency (33). As early as 2003, Sgourou found that the stability of mRNA containing four mutation sites in the 5′ UTR of the β-globin gene (*HBB*: c.-43C>T, *HBB*: c.-41delT, *HBB*: c.-29G>A, *HBB*: c.-11_-8delAACA) was reduced to varying degrees (34). However, research in recent years and data from our region indicated that the phenotype of *HBB*: c.-11_-8delAACA may be a benign variant (20), suggesting that the stability of mRNA at this site may not change significantly.

It is widely acknowledged that mRNA sequences are typically composed of permutations of four nucleotides (A, C, G, U). In base pairing interactions, AU and GC pairs form the most stable configurations through Watson-Crick base pairing, while GU pairs constitute less stable wobble base pairings (33). These nucleotide pairings serve as fundamental components of RNA secondary structure (35). The thermodynamic stability of Watson-Crick base pairs (GC > AU) significantly influences RNA structural formation (36), with GU wobble pairs providing structural flexibility while maintaining partial pairing stability (37). This structural organization plays crucial roles in post-transcriptional regulation, particularly in mRNA processing and translation initiation mechanisms (38). Using the RNAfold web server to predict the secondary structure of mRNA (15), we found that the secondary structure of mRNA with the *HBB*: c.-23A>G mutation did not significantly differ in stability compared to the normal sequence, and it did not alter the hairpin loop or stem-loop structures. The only change was that the alteration of this nucleotide caused the first internal loop of the mRNA to change from a stable Watson-Crick pair (AU) to a less stable wobble base pair (GU). This may be the reason why this variant has almost no impact on β-chain synthesis. In contrast, another mutation in this region (*HBB*: c.-29G>A), manifests as typical β^+^ thalassemia (39), likely because this mutation affects the hairpin loop of the mRNA.

A limitation of this study is the absence of functional assays, such as in vitro globin chain synthesis studies or luciferase reporter assays, to directly quantify the impact of the *HBB*: c.-23A>G mutation on translational efficiency or mRNA stability. Furthermore, the potential clinical consequence of co-inheritance with *α*-globin triplication remains unexplored. Previous reports have indicated that the coexistence of β-thalassemia and α-globin triplication can exacerbate the α/β-globin chain imbalance, potentially leading to a more severe anemic phenotype (40). In a prior investigation from our laboratory, 1,443 carriers of α-globin triplication were identified among 73,967 individuals in this region, yielding a carrier rate of 1.95% (41). Regrettably, due to the current sample size limitations, none of the 75 carriers of the *HBB*: c.-23A>G mutation in this study were found to co-inherit α-globin triplication. Consequently, we were unable to investigate the clinical manifestations of the combination of *HBB*: c.-23A>G and ααα. It is anticipated that future studies with larger sample sizes may identify such compound cases, allowing for a comprehensive assessment of their phenotypic impact.

## 5 Conclusion

In conclusion, we systematically characterized the clinical phenotype associated with the *HBB*: c.-23A>G mutation and elucidated the molecular rationale for its likely benign clinical manifestations. Additionally, comparative analysis of mRNA secondary structures demonstrated minimal thermodynamic destabilization compared to the wild-type sequence, with preserved hairpin loop and stem-loop architectures. Collectively, our findings provide valuable insights for improving genetic counseling for carriers of this mutation in clinical practice.

## Data Availability

The datasets presented in this study can be found in online repositories. The names of the repository/repositories and accession number(s) can be found in the article/Supplementary material. Raw sequencing data are available from the European Nucleotide Archive (ENA) under accession number PRJEB88162 (https://www.ebi.ac.uk/ena/browser/view/PRJEB88162).

## Footnotes

^1^ https://globin.bx.psu.edu/hbvar/

^2^ https://www.ithanet.eu/db/ithagenes
