# Supplementary material for: Molecular identification and phenotypic study of a novel HBB: c.-23A>G mutation in the 5’ untranslated region
Source: Front Med (Lausanne). 2025 Oct 30;12:1675600. doi: 10.3389/fmed.2025.1675600 (PMC12611677; doi:10.3389/fmed.2025.1675600)
Supplement: Supplementary file 1 [file Data_Sheet_1.docx]

Molecular Identification and Phenotypic Study of a Novel *HBB*: c.-23A>G Mutation in the 5’ Untranslated Region

Shichun Shen^1†^, Jungao Huang^1†^, Haimei Qi^2^, Zezhang Liu^3, 4^, Wenqian Zhang^3, 4^, Xianping Yuan^5^, Zhuling Zhang^5^, Haijun Chen^1^, Xinxing Xie^1^, Lin Xiao^1^, JunKun Chen^1^ and Liyun Song^6,^*

^1^Department of medical genetics, Ganzhou Maternal and Child Health Hospital, Ganzhou, China;

^2^Department of clinical Laboratory, Ganzhou Maternal and Child Health Hospital, Ganzhou, China;

^3^BGI Genomics, Shenzhen, China;

^4^Clin Lab, BGI Genomics, Wuhan, China;

^5^Obstetrical department, Ganzhou Maternal and Child Health Hospital, Ganzhou, China;

^6^Department of Clinical Laboratory, Ganzhou People's Hospital, Ganzhou, China;

*** Correspondence:**Liyun Song
[chinesu@126.com](mailto:chinesuzg@163.com)

† These authors contributed equally to this work.

Supplementary material

Table S1. Hematological Data of the Control Group. All cases in the control group had their genotypes clearly determined by NGS, and iron-deficiency anemia was excluded.

| Sex | Age | α-globin Genotype | β-globin Genotype | MCV (fL) | MCH (pg) | Hb A2 (%) |
| --- | --- | --- | --- | --- | --- | --- |
| Female | 39 | αα/αα | β*^HBB^*^:c.126_129delCTTT^/β^N^ | 62.6 | 20 | 5.3 |
| Female | 44 | αα/αα | β*^HBB^*^:c.126_129delCTTT^/β^N^ | 62.7 | 20.4 | 5.9 |
| Female | 25 | αα/αα | β*^HBB^*^:c.126_129delCTTT^/β^N^ | 60.9 | 19.5 | 5.2 |
| Female | 29 | αα/αα | β*^HBB^*^:c.126_129delCTTT^/β^N^ | 65.6 | 20.2 | 5.3 |
| Female | 30 | αα/αα | β*^HBB^*^:c.126_129delCTTT^/β^N^ | 62.7 | 19.7 | 5.5 |
| Female | 44 | αα/αα | β*^HBB^*^:c.126_129delCTTT^/β^N^ | 66.8 | 20.4 | 5.8 |
| Female | 20 | αα/αα | β*^HBB^*^:c.126_129delCTTT^/β^N^ | 65.9 | 20.5 | 4.9 |
| Female | 25 | αα/αα | β*^HBB^*^:c.126_129delCTTT^/β^N^ | 64.5 | 19.7 | 5.2 |
| Female | 20 | αα/αα | β*^HBB^*^:c.126_129delCTTT^/β^N^ | 66.4 | 20.6 | 5 |
| Female | 29 | αα/αα | β*^HBB^*^:c.126_129delCTTT^/β^N^ | 62.3 | 19.3 | 6.1 |
| Female | 43 | αα/αα | β*^HBB^*^:c.126_129delCTTT^/β^N^ | 64.2 | 22.6 | 5.3 |
| Female | 21 | αα/αα | β*^HBB^*^:c.126_129delCTTT^/β^N^ | 65.2 | 21.6 | 6.1 |
| Female | 21 | αα/αα | β*^HBB^*^:c.126_129delCTTT^/β^N^ | 65.2 | 21.6 | 6.1 |
| Female | 18 | αα/αα | β*^HBB^*^:c.126_129delCTTT^/β^N^ | 66.8 | 20.6 | 5.6 |
| Female | 30 | αα/αα | β*^HBB^*^:c.126_129delCTTT^/β^N^ | 65.6 | 21.5 | 5.5 |
| Female | 24 | αα/αα | β*^HBB^*^:c.126_129delCTTT^/β^N^ | 58.9 | 19.4 | 5.3 |
| Female | 34 | αα/αα | β*^HBB^*^:c.126_129delCTTT^/β^N^ | 59 | 19.6 | 5.6 |
| Female | 26 | αα/αα | β*^HBB^*^:c.126_129delCTTT^/β^N^ | 65.8 | 21.4 | 5.4 |
| Female | 31 | αα/αα | β*^HBB^*^:c.126_129delCTTT^/β^N^ | 64.4 | 21.2 | 4.6 |
| Female | 30 | αα/αα | β*^HBB^*^:c.126_129delCTTT^/β^N^ | 63.5 | 21.5 | 5.6 |
| Female | 24 | αα/αα | β*^HBB^*^:c.126_129delCTTT^/β^N^ | 63.8 | 19.7 | 5.4 |
| Female | 17 | αα/αα | β*^HBB^*^:c.126_129delCTTT^/β^N^ | 62.5 | 20.1 | 5.4 |
| Female | 22 | αα/αα | β*^HBB^*^:c.126_129delCTTT^/β^N^ | 63.9 | 21.2 | 5.4 |
| Female | 37 | αα/αα | β*^HBB^*^:c.126_129delCTTT^/β^N^ | 63.6 | 20 | 5 |
| Female | 20 | αα/αα | β*^HBB^*^:c.126_129delCTTT^/β^N^ | 64.6 | 21.4 | 5.7 |
| Female | 23 | αα/αα | β*^HBB^*^:c.126_129delCTTT^/β^N^ | 63.7 | 20.3 | 5.5 |
| Male | 28 | αα/αα | β*^HBB^*^:c.126_129delCTTT^/β^N^ | 66.1 | 19.8 | 4.7 |
| Male | 30 | αα/αα | β*^HBB^*^:c.126_129delCTTT^/β^N^ | 65.7 | 21.8 | 5.4 |
| Male | 33 | αα/αα | β*^HBB^*^:c.126_129delCTTT^/β^N^ | 63.4 | 20.4 | 5.3 |
| Male | 26 | αα/αα | β*^HBB^*^:c.126_129delCTTT^/β^N^ | 64.6 | 19.7 | 5.1 |
| Male | 29 | αα/αα | β*^HBB^*^:c.126_129delCTTT^/β^N^ | 61.8 | 19.8 | 6.1 |
| Male | 27 | αα/αα | β*^HBB^*^:c.126_129delCTTT^/β^N^ | 68 | 21.4 | 5.5 |
| Male | 35 | αα/αα | β*^HBB^*^:c.126_129delCTTT^/β^N^ | 61.1 | 19.4 | 5.4 |
| Male | 22 | αα/αα | β*^HBB^*^:c.126_129delCTTT^/β^N^ | 62.3 | 19.9 | 5 |
| Male | 28 | αα/αα | β*^HBB^*^:c.126_129delCTTT^/β^N^ | 57.9 | 17.3 | 5.3 |
| Male | 29 | αα/αα | β*^HBB^*^:c.126_129delCTTT^/β^N^ | 65.6 | 22.7 | 5.6 |
| Male | 24 | αα/αα | β*^HBB^*^:c.126_129delCTTT^/β^N^ | 64.1 | 19.7 | 5.7 |
| Male | 27 | αα/αα | β*^HBB^*^:c.126_129delCTTT^/β^N^ | 63.5 | 21.2 | 5.5 |
| Male | 32 | αα/αα | β*^HBB^*^:c.126_129delCTTT^/β^N^ | 64.6 | 21.3 | 5 |
| Male | 29 | αα/αα | β*^HBB^*^:c.126_129delCTTT^/β^N^ | 66.6 | 20.5 | 5.8 |
| Male | 31 | αα/αα | β*^HBB^*^:c.126_129delCTTT^/β^N^ | 63.6 | 20 | 5.1 |
| Male | 28 | αα/αα | β*^HBB^*^:c.126_129delCTTT^/β^N^ | 64.2 | 20.5 | 5.7 |
| Male | 28 | αα/αα | β*^HBB^*^:c.126_129delCTTT^/β^N^ | 63.5 | 20.5 | 5.1 |
| Male | 36 | αα/αα | β*^HBB^*^:c.126_129delCTTT^/β^N^ | 64.3 | 20.9 | 5.7 |
| Male | 24 | αα/αα | β*^HBB^*^:c.126_129delCTTT^/β^N^ | 62.5 | 20.5 | 5.8 |
| Male | 24 | αα/αα | β*^HBB^*^:c.126_129delCTTT^/β^N^ | 59.4 | 20.2 | 5.9 |
| Male | 33 | αα/αα | β*^HBB^*^:c.126_129delCTTT^/β^N^ | 63.7 | 18.6 | 6 |
| Male | 36 | αα/αα | β*^HBB^*^:c.126_129delCTTT^/β^N^ | 63 | 20.7 | 6.3 |
| Male | 28 | αα/αα | β*^HBB^*^:c.126_129delCTTT^/β^N^ | 57.9 | 17.3 | 5.3 |
| Male | 44 | αα/αα | β*^HBB^*^:c.126_129delCTTT^/β^N^ | 63.4 | 19.6 | 5.4 |
| Male | 31 | αα/αα | β*^HBB^*^:c.126_129delCTTT^/β^N^ | 60.8 | 20.2 | 4.9 |
| Male | 33 | αα/αα | β*^HBB^*^:c.126_129delCTTT^/β^N^ | 62.9 | 18.5 | 5.2 |
| Male | 22 | αα/αα | β*^HBB^*^:c.126_129delCTTT^/β^N^ | 66.9 | 20.8 | 5.8 |
| Male | 23 | αα/αα | β*^HBB^*^:c.126_129delCTTT^/β^N^ | 63.3 | 21 | 5 |
| Male | 34 | αα/αα | β*^HBB^*^:c.126_129delCTTT^/β^N^ | 64.6 | 21.3 | 5.4 |
| Male | 28 | αα/αα | β*^HBB^*^:c.126_129delCTTT^/β^N^ | 60.6 | 19.6 | 5.5 |
| Female | 23 | αα/αα | β*^HBB^*^:c.126_129delCTTT^/β^N^ | 63.2 | 19.8 | 5.3 |
| Female | 26 | αα/αα | β*^HBB^*^:c.126_129delCTTT^/β^N^ | 64.3 | 20.8 | 5.1 |
| Female | 28 | αα/αα | β*^HBB^*^:c.126_129delCTTT^/β^N^ | 60.4 | 19.4 | 5.4 |
| Female | 31 | αα/αα | β*^HBB^*^:c.126_129delCTTT^/β^N^ | 62.3 | 19.7 | 5.8 |
| Male | 26 | αα/αα | β*^HBB^*^:c.-78A>G^/β^N^ | 69.1 | 21.5 | 5.9 |
| Female | 24 | αα/αα | β*^HBB^*^:c.-78A>G^/β^N^ | 69.8 | 21.4 | 5.5 |
| Male | 27 | αα/αα | β*^HBB^*^:c.-78A>G^/β^N^ | 72.3 | 23.7 | 5.5 |
| Male | 27 | αα/αα | β*^HBB^*^:c.-78A>G^/β^N^ | 68.6 | 22.5 | 6.2 |
| Female | 22 | αα/αα | β*^HBB^*^:c.-78A>G^/β^N^ | 72.9 | 24 | 6 |
| Female | 28 | αα/αα | β*^HBB^*^:c.-78A>G^/β^N^ | 73.3 | 23.3 | 6.2 |
| Female | 25 | αα/αα | β*^HBB^*^:c.-78A>G^/β^N^ | 73.2 | 22.6 | 5.5 |
| Female | 31 | αα/αα | β*^HBB^*^:c.-78A>G^/β^N^ | 73.2 | 23.3 | 5.6 |
| Male | 54 | αα/αα | β*^HBB^*^:c.-78A>G^/β^N^ | 73.4 | 24.1 | 5.2 |
| Male | 35 | αα/αα | β*^HBB^*^:c.-78A>G^/β^N^ | 74.4 | 21.3 | 5.1 |
| Female | 26 | αα/αα | β*^HBB^*^:c.-78A>G^/β^N^ | 72.7 | 24.8 | 5.9 |
| Male | 22 | αα/αα | β*^HBB^*^:c.-78A>G^/β^N^ | 74.2 | 24.2 | 6 |
| Female | 28 | αα/αα | β*^HBB^*^:c.-78A>G^/β^N^ | 71.5 | 23.2 | 6.3 |
| Male | 36 | αα/αα | β*^HBB^*^:c.-78A>G^/β^N^ | 69.8 | 22.1 | 5.8 |
| Female | 31 | αα/αα | β*^HBB^*^:c.-78A>G^/β^N^ | 71.4 | 24.5 | 5.1 |
| Female | 41 | αα/αα | β*^HBB^*^:c.-78A>G^/β^N^ | 70.7 | 23 | 5.1 |
| Male | 25 | αα/αα | β*^HBB^*^:c.-78A>G^/β^N^ | 64.2 | 21.5 | 5.4 |
| Female | 30 | αα/αα | β*^HBB^*^:c.-78A>G^/β^N^ | 65.8 | 23.4 | 5.6 |
| Male | 46 | αα/αα | β*^HBB^*^:c.-78A>G^/β^N^ | 68.5 | 22.5 | 5.7 |
| Male | 31 | αα/αα | β*^HBB^*^:c.-78A>G^/β^N^ | 75.3 | 23.6 | 5.8 |
| Male | 41 | αα/αα | β*^HBB^*^:c.-78A>G^/β^N^ | 74 | 24 | 5.7 |
| Female | 25 | αα/αα | β*^HBB^*^:c.-78A>G^/β^N^ | 70.4 | 23.1 | 5.7 |
| Female | 19 | αα/αα | β*^HBB^*^:c.-78A>G^/β^N^ | 66.5 | 22 | 5.7 |
| Male | 31 | αα/αα | β*^HBB^*^:c.-78A>G^/β^N^ | 74.3 | 20.8 | 5.1 |
| Female | 27 | αα/αα | β*^HBB^*^:c.-78A>G^/β^N^ | 70.4 | 21 | 4.5 |
| Female | 24 | αα/αα | β*^HBB^*^:c.-78A>G^/β^N^ | 71.4 | 22.7 | 4.7 |
| Female | 30 | αα/αα | β*^HBB^*^:c.-78A>G^/β^N^ | 69.4 | 22.5 | 5.7 |
| Male | 35 | αα/αα | β*^HBB^*^:c.-78A>G^/β^N^ | 74.3 | 24.5 | 5.3 |
| Male | 26 | αα/αα | β*^HBB^*^:c.-78A>G^/β^N^ | 70.6 | 22.3 | 5.2 |
| Male | 22 | αα/αα | β*^HBB^*^:c.-78A>G^/β^N^ | 73.2 | 23.8 | 5.5 |
| Female | 33 | αα/αα | β*^HBB^*^: c.-11_-8delAACA^/β^N^ | 85 | 28.2 | 2.5 |
| Female | 31 | αα/αα | β*^HBB^*^: c.-11_-8delAACA^/β^N^ | 95.6 | 31.3 | 2.8 |
| Male | 31 | αα/αα | β*^HBB^*^: c.-11_-8delAACA^/β^N^ | 95.8 | 31.8 | 2.6 |
| Female | 26 | αα/αα | β*^HBB^*^: c.-11_-8delAACA^/β^N^ | 93 | 28.6 | 2.6 |
| Male | 25 | αα/αα | β*^HBB^*^: c.-11_-8delAACA^/β^N^ | 85.7 | 29.2 | 3 |
| Male | 32 | αα/αα | β*^HBB^*^: c.-11_-8delAACA^/β^N^ | 92.3 | 30.1 | 2.6 |
| Female | 26 | αα/αα | β*^HBB^*^: c.-11_-8delAACA^/β^N^ | 89.8 | 29.3 | 2.7 |
| Female | 19 | αα/αα | β*^HBB^*^: c.-11_-8delAACA^/β^N^ | 93.2 | 31.1 | 2.6 |
| Male | 23 | αα/αα | β*^HBB^*^: c.-11_-8delAACA^/β^N^ | 95.4 | 32.1 | 2.7 |
| Female | 33 | αα/αα | β*^HBB^*^: c.-11_-8delAACA^/β^N^ | 88.6 | 28.9 | 2.8 |
| Male | 29 | αα/αα | β*^HBB^*^: c.-11_-8delAACA^/β^N^ | 91.9 | 30.1 | 2.6 |
| Male | 32 | αα/αα | β*^HBB^*^: c.-11_-8delAACA^/β^N^ | 92.2 | 30.6 | 2.7 |
| Male | 23 | αα/αα | β*^HBB^*^: c.-11_-8delAACA^/β^N^ | 90.9 | 30.7 | 2.7 |
| Male | 34 | αα/αα | β*^HBB^*^: c.-11_-8delAACA^/β^N^ | 92 | 32.1 | 2.6 |
| Male | 34 | αα/αα | β*^HBB^*^: c.-11_-8delAACA^/β^N^ | 99.2 | 33.1 | 2.7 |
| Male | 34 | αα/αα | β*^HBB^*^: c.-11_-8delAACA^/β^N^ | 90.1 | 29.6 | 2.9 |
| Female | 32 | αα/αα | β*^HBB^*^: c.-11_-8delAACA^/β^N^ | 93.1 | 32.6 | 2.8 |
| Female | 23 | αα/αα | β*^HBB^*^: c.-11_-8delAACA^/β^N^ | 100.2 | 33.3 | 2.8 |
| Female | 29 | αα/αα | β*^HBB^*^: c.-11_-8delAACA^/β^N^ | 91.6 | 30.9 | 2.5 |
| Female | 21 | αα/αα | β*^HBB^*^: c.-11_-8delAACA^/β^N^ | 90.8 | 29.4 | 2.8 |
| Female | 27 | αα/αα | β*^HBB^*^: c.-29G>A^/β^N^ | 72.7 | 23.7 | 4.1 |
| Female | 23 | αα/αα | β*^HBB^*^: c.-29G>A^/β^N^ | 73 | 24 | 4.2 |
| Female | 27 | αα/αα | β*^HBB^*^: c.-29G>A^/β^N^ | 75.3 | 23.8 | 3.9 |
| Female | 23 | αα/αα | β*^HBB^*^: c.-29G>A^/β^N^ | 74.3 | 23.8 | 4.1 |
| Female | 23 | αα/αα | β*^HBB^*^: c.-29G>A^/β^N^ | 73.8 | 24.3 | 4.5 |
| Male | 25 | αα/αα | β*^HBB^*^: c.-29G>A^/β^N^ | 73 | 24.3 | 4.2 |
| Male | 25 | αα/αα | β*^HBB^*^: c.-29G>A^/β^N^ | 74.6 | 24.6 | 4 |
| Male | 24 | αα/αα | β*^HBB^*^: c.-29G>A^/β^N^ | 72.8 | 23.7 | 3.9 |
| Male | 23 | αα/αα | β*^HBB^*^: c.-29G>A^/β^N^ | 71.9 | 23.5 | 4.3 |
| Male | 32 | αα/αα | β*^HBB^*^: c.-29G>A^/β^N^ | 71.6 | 23.3 | 4.1 |
| Male | 38 | αα/αα | β^N^/β^N^ | 96.8 | 30.9 | 2.9 |
| Male | 31 | αα/αα | β^N^/β^N^ | 88.2 | 29.1 | 2.6 |
| Male | 34 | αα/αα | β^N^/β^N^ | 91.2 | 29.7 | 2.5 |
| Male | 28 | αα/αα | β^N^/β^N^ | 88.8 | 28.7 | 2.4 |
| Male | 31 | αα/αα | β^N^/β^N^ | 91.3 | 29.7 | 2.7 |
| Male | 30 | αα/αα | β^N^/β^N^ | 90.2 | 29.4 | 2.4 |
| Male | 33 | αα/αα | β^N^/β^N^ | 88.2 | 27.7 | 2.3 |
| Male | 34 | αα/αα | β^N^/β^N^ | 95.8 | 31.2 | 2.6 |
| Male | 38 | αα/αα | β^N^/β^N^ | 94.4 | 32.3 | 2.7 |
| Male | 28 | αα/αα | β^N^/β^N^ | 86.5 | 28.2 | 2.6 |
| Male | 23 | αα/αα | β^N^/β^N^ | 92.4 | 29.8 | 2.6 |
| Female | 30 | αα/αα | β^N^/β^N^ | 95.8 | 32 | 2.8 |
| Female | 31 | αα/αα | β^N^/β^N^ | 92.9 | 30.9 | 2.8 |
| Female | 21 | αα/αα | β^N^/β^N^ | 92 | 31 | 2.7 |
| Female | 34 | αα/αα | β^N^/β^N^ | 92.8 | 30.9 | 2.5 |
| Female | 27 | αα/αα | β^N^/β^N^ | 96.3 | 31.5 | 2.9 |
| Female | 37 | αα/αα | β^N^/β^N^ | 86.9 | 29.2 | 2.9 |
| Female | 25 | αα/αα | β^N^/β^N^ | 89 | 29.1 | 2.7 |
| Female | 23 | αα/αα | β^N^/β^N^ | 90.1 | 30 | 3.1 |
| Female | 26 | αα/αα | β^N^/β^N^ | 94.4 | 31.8 | 2.7 |
| Female | 26 | αα/αα | β^N^/β^N^ | 92.6 | 29.5 | 2.5 |
| Female | 29 | αα/αα | β^N^/β^N^ | 92.1 | 30.4 | 2.9 |
| Female | 27 | αα/αα | β^N^/β^N^ | 90.2 | 30 | 2.5 |
| Female | 33 | αα/αα | β^N^/β^N^ | 88.8 | 29.4 | 2.4 |
| Female | 35 | αα/αα | β^N^/β^N^ | 89.2 | 29.5 | 2.5 |
| Female | 31 | αα/αα | β^N^/β^N^ | 95 | 30.7 | 2.9 |
| Female | 22 | αα/αα | β^N^/β^N^ | 92.4 | 31.2 | 2.6 |
| Female | 24 | αα/αα | β^N^/β^N^ | 93.5 | 31 | 2.7 |
| Female | 23 | αα/αα | β^N^/β^N^ | 89.3 | 29.7 | 2.5 |
| Female | 28 | αα/αα | β^N^/β^N^ | 89.1 | 29.8 | 2.5 |
| Female | 33 | αα/αα | β^N^/β^N^ | 89.9 | 30.3 | 2.6 |
| Female | 35 | αα/αα | β^N^/β^N^ | 91.6 | 31 | 2.6 |
| Female | 26 | αα/αα | β^N^/β^N^ | 92.8 | 30.8 | 2.5 |
| Female | 23 | αα/αα | β^N^/β^N^ | 95 | 29.8 | 2.6 |
| Female | 36 | αα/αα | β^N^/β^N^ | 98.9 | 31.4 | 2.5 |
| Female | 31 | αα/αα | β^N^/β^N^ | 91.9 | 30.1 | 2.9 |
| Female | 28 | αα/αα | β^N^/β^N^ | 92.3 | 30.3 | 2.8 |
| Female | 34 | αα/αα | β^N^/β^N^ | 95.3 | 31.9 | 2.4 |
| Female | 33 | αα/αα | β^N^/β^N^ | 85.9 | 27.2 | 2.5 |
| Female | 28 | αα/αα | β^N^/β^N^ | 92.5 | 31.2 | 3.1 |
| Female | 26 | αα/αα | β^N^/β^N^ | 99.4 | 33.2 | 2.9 |
| Female | 33 | αα/αα | β^N^/β^N^ | 97.1 | 32.3 | 2.7 |
| Female | 23 | αα/αα | β^N^/β^N^ | 92.3 | 31.8 | 3 |
| Female | 24 | αα/αα | β^N^/β^N^ | 94 | 31.2 | 3.2 |
| Female | 28 | αα/αα | β^N^/β^N^ | 95 | 31 | 2.8 |
| Female | 26 | αα/αα | β^N^/β^N^ | 88.6 | 29 | 3.1 |
| Female | 27 | αα/αα | β^N^/β^N^ | 91.5 | 30 | 2.7 |
| Female | 33 | αα/αα | β^N^/β^N^ | 94.3 | 31.1 | 2.8 |
| Female | 28 | αα/αα | β^N^/β^N^ | 94.5 | 30.9 | 2.6 |
| Female | 28 | αα/αα | β^N^/β^N^ | 95.2 | 31.1 | 2.9 |
| Female | 29 | αα/αα | β^N^/β^N^ | 94.3 | 30.8 | 2.6 |
| Male | 34 | αα/αα | β^N^/β^N^ | 90.1 | 29.6 | 2.9 |
| Male | 25 | αα/αα | β^N^/β^N^ | 96 | 30.5 | 3.1 |
| Male | 27 | αα/αα | β^N^/β^N^ | 89.6 | 29.7 | 2.7 |
| Male | 32 | αα/αα | β^N^/β^N^ | 88.1 | 30.3 | 2.5 |
| Male | 26 | αα/αα | β^N^/β^N^ | 92.5 | 31.1 | 2.6 |
| Male | 34 | αα/αα | β^N^/β^N^ | 97.3 | 32.4 | 2.7 |
| Male | 25 | αα/αα | β^N^/β^N^ | 90.3 | 29.7 | 2.5 |
| Male | 23 | αα/αα | β^N^/β^N^ | 89.8 | 31.5 | 2.8 |
| Male | 28 | αα/αα | β^N^/β^N^ | 89.6 | 28.9 | 2.7 |
| Male | 27 | αα/αα | β^N^/β^N^ | 87.7 | 29.3 | 2.9 |
| Male | 31 | αα/αα | β^N^/β^N^ | 92.5 | 31.9 | 2.5 |
| Male | 32 | αα/αα | β^N^/β^N^ | 89.5 | 30.6 | 2.6 |
| Male | 26 | αα/αα | β^N^/β^N^ | 90.3 | 29.1 | 2.7 |
| Male | 27 | αα/αα | β^N^/β^N^ | 88.9 | 29.1 | 2.8 |
| Male | 26 | αα/αα | β^N^/β^N^ | 97.3 | 32 | 2.5 |
| Male | 24 | αα/αα | β^N^/β^N^ | 97.3 | 28.3 | 2.6 |
| Male | 33 | αα/αα | β^N^/β^N^ | 91 | 31.8 | 2.8 |
| Male | 34 | αα/αα | β^N^/β^N^ | 91.2 | 29.4 | 2.6 |
| Male | 31 | αα/αα | β^N^/β^N^ | 86.1 | 29 | 2.7 |
| Male | 25 | αα/αα | β^N^/β^N^ | 93.3 | 32.3 | 2.9 |
| Male | 29 | αα/αα | β^N^/β^N^ | 91.9 | 32.9 | 2.4 |
| Male | 28 | αα/αα | β^N^/β^N^ | 89.7 | 31.6 | 2.6 |
| Male | 24 | αα/αα | β^N^/β^N^ | 86.3 | 28.1 | 2.8 |
| Male | 23 | αα/αα | β^N^/β^N^ | 92.3 | 30.6 | 2.7 |
| Male | 39 | αα/αα | β^N^/β^N^ | 97.1 | 32.1 | 3.1 |
| Male | 33 | αα/αα | β^N^/β^N^ | 95.3 | 31.7 | 2.7 |
| Male | 36 | αα/αα | β^N^/β^N^ | 93.9 | 30.5 | 2.9 |
| Male | 31 | αα/αα | β^N^/β^N^ | 96.1 | 32.1 | 2.7 |
| Male | 27 | αα/αα | β^N^/β^N^ | 98.3 | 33.3 | 2.8 |
| Female | 20 | αα/--^SEA^ | β^N^/β^N^ |  |  | 2.4 |
| Female | 40 | αα/--^SEA^ | β^N^/β^N^ |  |  | 2.3 |
| Female | 43 | αα/--^SEA^ | β^N^/β^N^ |  |  | 2.1 |
| Female | 23 | αα/--^SEA^ | β^N^/β^N^ |  |  | 2.2 |
| Female | 44 | αα/--^SEA^ | β^N^/β^N^ |  |  | 2.4 |
| Female | 43 | αα/--^SEA^ | β^N^/β^N^ |  |  | 2.5 |
| Female | 29 | αα/--^SEA^ | β^N^/β^N^ |  |  | 2.1 |
| Female | 26 | αα/--^SEA^ | β^N^/β^N^ |  |  | 2.4 |
| Female | 35 | αα/--^SEA^ | β^N^/β^N^ |  |  | 2.2 |
| Female | 30 | αα/--^SEA^ | β^N^/β^N^ |  |  | 2.4 |
| Female | 28 | αα/--^SEA^ | β^N^/β^N^ |  |  | 2.5 |
| Female | 25 | αα/--^SEA^ | β^N^/β^N^ |  |  | 2.5 |
| Female | 42 | αα/--^SEA^ | β^N^/β^N^ |  |  | 2.4 |
| Female | 30 | αα/--^SEA^ | β^N^/β^N^ |  |  | 2.5 |
| Female | 31 | αα/--^SEA^ | β^N^/β^N^ |  |  | 2.5 |
| Male | 27 | αα/--^SEA^ | β^N^/β^N^ |  |  | 2.4 |
| Male | 29 | αα/--^SEA^ | β^N^/β^N^ |  |  | 2.1 |
| Male | 23 | αα/--^SEA^ | β^N^/β^N^ |  |  | 2 |
| Male | 37 | αα/--^SEA^ | β^N^/β^N^ |  |  | 2.1 |
| Male | 32 | αα/--^SEA^ | β^N^/β^N^ |  |  | 2.2 |
| Male | 25 | αα/--^SEA^ | β^N^/β^N^ |  |  | 2.4 |
| Male | 23 | αα/--^SEA^ | β^N^/β^N^ |  |  | 2.4 |
| Male | 31 | αα/--^SEA^ | β^N^/β^N^ |  |  | 2.2 |
| Male | 27 | αα/--^SEA^ | β^N^/β^N^ |  |  | 2.2 |
| Male | 27 | αα/--^SEA^ | β^N^/β^N^ |  |  | 2.5 |
| Male | 29 | αα/--^SEA^ | β^N^/β^N^ |  |  | 2 |
| Male | 31 | αα/--^SEA^ | β^N^/β^N^ |  |  | 2.2 |
| Male | 29 | αα/--^SEA^ | β^N^/β^N^ |  |  | 2.5 |
| Male | 25 | αα/--^SEA^ | β^N^/β^N^ |  |  | 2.4 |
| Male | 27 | αα/--^SEA^ | β^N^/β^N^ |  |  | 2 |
| Male | 28 | αα/--^SEA^ | β*^HBB^*^:c.126_129delCTTT^/β^N^ |  |  | 4.9 |
| Female | 30 | αα/--^SEA^ | β*^HBB^*^:c.126_129delCTTT^/β^N^ |  |  | 6.2 |
| Female | 33 | αα/--^SEA^ | β*^HBB^*^:c.126_129delCTTT^/β^N^ |  |  | 5.3 |
| Male | 26 | αα/--^SEA^ | β*^HBB^*^:c.126_129delCTTT^/β^N^ |  |  | 5.7 |
| Female | 29 | αα/--^SEA^ | β*^HBB^*^:c.126_129delCTTT^/β^N^ |  |  | 5.1 |
| Male | 27 | αα/--^SEA^ | β*^HBB^*^:c.126_129delCTTT^/β^N^ |  |  | 5.4 |
| Female | 35 | αα/--^SEA^ | β*^HBB^*^:c.126_129delCTTT^/β^N^ |  |  | 5 |
| Male | 22 | αα/--^SEA^ | β*^HBB^*^:c.126_129delCTTT^/β^N^ |  |  | 5.1 |
| Female | 28 | αα/--^SEA^ | β*^HBB^*^:c.126_129delCTTT^/β^N^ |  |  | 5.8 |
| Male | 29 | αα/--^SEA^ | β*^HBB^*^:c.126_129delCTTT^/β^N^ |  |  | 5.7 |
| Male | 24 | αα/--^SEA^ | β*^HBB^*^:c.126_129delCTTT^/β^N^ |  |  | 4.9 |
| Female | 27 | αα/--^SEA^ | β*^HBB^*^:c.126_129delCTTT^/β^N^ |  |  | 5.5 |
| Male | 32 | αα/--^SEA^ | β*^HBB^*^:c.126_129delCTTT^/β^N^ |  |  | 5.2 |
| Male | 29 | αα/--^SEA^ | β*^HBB^*^:c.126_129delCTTT^/β^N^ |  |  | 4.6 |
| Female | 31 | αα/--^SEA^ | β*^HBB^*^:c.126_129delCTTT^/β^N^ |  |  | 4.5 |
| Male | 28 | αα/--^SEA^ | β*^HBB^*^:c.126_129delCTTT^/β^N^ |  |  | 4.9 |
| Female | 28 | αα/--^SEA^ | β*^HBB^*^:c.126_129delCTTT^/β^N^ |  |  | 4.5 |
| Male | 36 | αα/--^SEA^ | β*^HBB^*^:c.126_129delCTTT^/β^N^ |  |  | 4.3 |
| Female | 24 | αα/--^SEA^ | β*^HBB^*^:c.126_129delCTTT^/β^N^ |  |  | 4.5 |
| Male | 24 | αα/--^SEA^ | β*^HBB^*^:c.126_129delCTTT^/β^N^ |  |  | 5.3 |
| Female | 26 | αα/--^SEA^ | β*^HBB^*^:c.-78A>G^/β^N^ |  |  | 5.1 |
| Female | 27 | αα/--^SEA^ | β*^HBB^*^:c.-78A>G^/β^N^ |  |  | 5.1 |
| Female | 27 | αα/--^SEA^ | β*^HBB^*^:c.-78A>G^/β^N^ |  |  | 6.4 |
| Female | 54 | αα/--^SEA^ | β*^HBB^*^:c.-78A>G^/β^N^ |  |  | 5.6 |
| Male | 35 | αα/--^SEA^ | β*^HBB^*^:c.-78A>G^/β^N^ |  |  | 5.7 |
| Male | 22 | αα/--^SEA^ | β*^HBB^*^:c.-78A>G^/β^N^ |  |  | 5.8 |
| Female | 36 | αα/--^SEA^ | β*^HBB^*^:c.-78A>G^/β^N^ |  |  | 5.7 |
| Male | 25 | αα/--^SEA^ | β*^HBB^*^:c.-78A>G^/β^N^ |  |  | 5.7 |
| Female | 46 | αα/--^SEA^ | β*^HBB^*^:c.-78A>G^/β^N^ |  |  | 5.7 |
| Female | 31 | αα/--^SEA^ | β*^HBB^*^:c.-78A>G^/β^N^ |  |  | 5.1 |

**Table S2.** Clinical Information and Hematological Data of 75 Carriers with the *HBB*: c.-23A>G Mutation.

| Case ID | Sex | Age | α-globin Genotype | β-globin Genotype | RBC (10^12^/L) | HB (g/L) | MCV (fL) | MCH (pg) | Ferritin (ug/L) | Hb A (%) | Hb A2 (%) | Hb F (%) | Notes |
| --- | --- | --- | --- | --- | --- | --- | --- | --- | --- | --- | --- | --- | --- |
| 1 | Female | 26 | αα/αα | β*^HBB^*^:c.-23A>G^/β^N^ | 3.77 | 115 | 94.8 | 30.4 | 80.8 | 97.1 | 2.9 |  |  |
| 2 | Female | 23 | αα/αα | β*^HBB^*^:c.-23A>G^/β^N^ | 3.46 | 111 | 98.6 | 32.2 | 50.5 | 97.1 | 2.9 |  |  |
| 3 | Female | 33 | αα/αα | β*^HBB^*^:c.-23A>G^/β^N^ | 3.70 | 118 | 96.7 | 31.8 | 33.9 | 97.4 | 2.6 |  |  |
| 4 | Male | 40 | αα/αα | β*^HBB^*^:c.-23A>G^/β^N^ | 5.33 | 167 | 92.9 | 31.3 |  | 97.6 | 2.4 |  |  |
| 5 | Female | 28 | αα/αα | β*^HBB^*^:c.-23A>G^/β^N^ | 3.76 | 119 | 96.9 | 31.7 | 16.9 | 97.3 | 2.3 | 0.4 |  |
| 6 | Female | 32 | αα/αα | β*^HBB^*^:c.-23A>G^/β^N^ | 3.19 | 111 | 99.5 | 34.8 | 33.9 | 97.3 | 2.7 |  |  |
| 7 | Female | 24 | αα/αα | β*^HBB^*^:c.-23A>G^/β^N^ | 4.22 | 132 | 89.9 | 31.3 | 10.2^a^ | 97.5 | 2.5 |  |  |
| 8^b^ | Female | 29 | αα/αα | β*^HBB^*^:c.-23A>G^/β^N^ | 3.89 | 124 | 91.2 | 31.8 | 37.7 | 96.0 | 2.8 | 1.2 |  |
| 9^c^ | Male | 33 | αα/αα | β*^HBB^*^:c.-23A>G^/β^N^ | 5.30 | 157 | 97.9 | 29.6 |  | 97.4 | 2.6 |  | Wife's genotype: αα/αα β*^HBB^*^:c.316-197C>T^/β^N^. |
| 10 | Female | 25 | αα/--^SEA^ | β*^HBB^*^:c.-23A>G^/β^N^ | 5.51 | 121 | 71.2 | 21.9 | 64.9 | 97.7 | 2.3 |  |  |
| 11 | Female | 27 | αα/αα | β*^HBB^*^:c.-23A>G^/β^N^ | 4.01 | 126 | 99.8 | 31.5 | 46.3 | 97.3 | 2.7 |  |  |
| 12 | Female | 40 | αα/αα | β*^HBB^*^:c.-23A>G^/β^N^ | 4.27 | 127 | 87.3 | 29.8 |  | 97.2 | 2.8 |  |  |
| 13 | Female | 31 | αα/--^SEA^ | β*^HBB^*^:c.-23A>G^/β^N^ | 5.24 | 114 | 73.4 | 21.8 | 28.2 | 97.7 | 2.3 |  |  |
| 14^c^ | Female | 28 | αα/αα | β*^HBB^*^:c.-23A>G^/β^N^ | 4.77 | 148 | 96.3 | 31.0 | 55.1 | 97.6 | 2.4 |  | Husband's genotype: αα/αα β*^HBB^*^:c.316-197C>T^/β^N^. |
| 15 | Male | 36 | αα/--^SEA^ | β*^HBB^*^:c.-23A>G^/β^N^ | 5.36 | 110 | 69.5 | 20.4 |  | 97.8 | 2.2 |  |  |
| 16 | Female | 30 | αα/αα | β*^HBB^*^:c.-23A>G^/β^N^ | 4.19 | 115 | 84.8 | 27.5 | 40.4 | 97.4 | 2.6 |  |  |
| 17 | Female | 23 | αα/αα | β*^HBB^*^:c.-23A>G^/β^N^ | 5.08 | 147 | 88.9 | 29.0 | 70.4 | 97.3 | 2.7 |  |  |
| 18^b^ | Female | 26 | αα/αα | β*^HBB^*^:c.-23A>G^/β^N^ | 4.38 | 141 | 92.9 | 32.3 | 30.5 | 96.7 | 2.7 | 0.6 |  |
| 19 | Male | 27 | αα/αα | β*^HBB^*^:c.-23A>G^/β^N^ | 5.10 | 159 | 93.2 | 31.2 |  | 97.5 | 2.5 |  |  |

| 20 | Female | 30 | αα/αα | β*^HBB^*^:c.-23A>G^/β^N^ | 4.23 | 123 | 89.7 | 29.0 | 39.2 | 97.1 | 2.9 |  |  |
| --- | --- | --- | --- | --- | --- | --- | --- | --- | --- | --- | --- | --- | --- |
| 21 | Female | 31 | αα/αα | β*^HBB^*^:c.-23A>G^/β*^HBB^*^:c.316-197C>T^ | 5.80 | 125 | 68.8 | 21.6 | 41.3 | 95.2 | 4.8 |  |  |
| 22 | Female | 25 | αα/αα | β*^HBB^*^:c.-23A>G^/β^N^ | 3.59 | 111 | 93.8 | 30.8 | 37 | 97.4 | 2.6 |  |  |
| 23 | Female | 28 | αα/--^SEA^ | β*^HBB^*^:c.-23A>G^/β^N^ | 5.15 | 116 | 70.5 | 22.5 | 8.93^a^ | 97.6 | 2.4 |  |  |
| 24 | Female | 23 | αα/αα | β*^HBB^*^:c.-23A>G^/β^N^ | 4.23 | 128 | 90.3 | 30.3 | 27.3 | 97.3 | 2.7 |  |  |
| 25 | Female | 25 | αα/αα | β*^HBB^*^:c.-23A>G^/β^N^ | 4.20 | 130 | 93.0 | 31.0 | 17.6 | 97.4 | 2.6 |  |  |
| 26 | Female | 33 | αα/αα | β*^HBB^*^:c.-23A>G^/β^N^ | 3.59 | 111 | 96.1 | 31.0 | 15.5 | 97.3 | 2.7 |  |  |
| 27 | Female | 28 | αα/αα | β*^HBB^*^:c.-23A>G^/β^N^ | 4.17 | 130 | 93.4 | 31.1 | 25.3 | 97.2 | 2.8 |  |  |
| 28 | Female | 24 | αα/αα | β*^HBB^*^:c.-23A>G^/β^N^ | 4.62 | 118 | 75.8 | 25.5 | 8.6a | 97.6 | 2.4 |  |  |
| 29 | Female | 22 | αα/αα | β*^HBB^*^:c.-23A>G^/β^N^ | 4.25 | 130 | 92.4 | 30.6 | 46.2 | 97.5 | 2.5 |  |  |
| 30 | Female | 31 | αα/αα | β*^HBB^*^:c.-23A>G^/β^N^ | 4.02 | 125 | 94.1 | 31.1 | 37.9 | 97.4 | 2.6 |  |  |
| 31 | Female | 38 | αα/--SEA | β*^HBB^*^:c.-23A>G^/β^N^ | 5.48 | 120 | 69.4 | 22.0 | 56.7 | 97.6 | 2.4 |  |  |
| 32^c^ | Female | 20 | αα/αα | β*^HBB^*^:c.-23A>G^/β^N^ | 3.83 | 116 | 90.8 | 30.2 | 16.6 | 97.4 | 2.6 |  | Husband's genotype: αα/αα β*^HBB^*^:c.-78A>C^/β^N^. |
| 33 | Female | 24 | αα/αα | β*^HBB^*^:c.-23A>G^/β^N^ | 3.80 | 118 | 93.9 | 31.0 | 112 | 97.0 | 3.0 |  |  |
| 34 | Female | 26 | αα/αα | β*^HBB^*^:c.-23A>G^/β^N^ | 4.27 | 129 | 94.3 | 30.3 | 23 | 97.0 | 3.0 |  |  |
| 35 | Female | 20 | αα/αα | β*^HBB^*^:c.-23A>G^/β^N^ | 4.49 | 103 | 72.0 | 22.9 | 6.9^a^ | 97.7 | 2.3 |  |  |
| 36 | Female | 20 | αα/αα | β*^HBB^*^:c.-23A>G^/β^N^ | 4.10 | 144 | 97.8 | 35.1 | 14.7 | 97.3 | 2.7 |  |  |
| 37 | Male | 25 | αα/αα | β*^HBB^*^:c.-23A>G^/β^N^ | 4.96 | 153 | 88.3 | 30.9 |  | 97.4 | 2.6 |  |  |
| 38 | Female | 20 | αα/αα | β*^HBB^*^:c.-23A>G^/β^N^ | 3.59 | 118 | 99.1 | 32.7 | 35.3 | 97.1 | 2.9 |  |  |
| 39 | Male | 23 | αα/αα | β*^HBB^*^:c.-23A>G^/β^N^ | 5.47 | 162 | 85.3 | 29.7 |  | 97.2 | 2.8 |  |  |
| 40 | Male | 23 | αα/αα | β*^HBB^*^:c.-23A>G^/β^N^ | 4.82 | 151 | 95.4 | 31.3 | 68.2 | 97.0 | 3.0 |  |  |
| 41 | Male | 24 | αα/αα | β*^HBB^*^:c.-23A>G^/β^N^ | 5.45 | 159 | 89.1 | 29.1 | 18.6 | 97.5 | 2.5 |  |  |
| 42 | Male | 36 | αα/αα | β*^HBB^*^:c.-23A>G^/β^N^ | 5.41 | 154 | 88.8 | 28.5 |  | 97.4 | 2.6 |  |  |
| 43 | Male | 35 | αα/αα | β*^HBB^*^:c.-23A>G^/β^N^ | 4.56 | 132 | 91.4 | 28.9 | 27.2 | 97.4 | 2.6 |  |  |
| 44 | Male | 26 | αα/αα | β*^HBB^*^:c.-23A>G^/β^N^ | 4.73 | 151 | 94.4 | 31.9 | 53.2 | 97.5 | 2.5 |  |  |
| 45 | Female | 23 | αα/αα | β*^HBB^*^:c.-23A>G^/β^N^ | 3.98 | 121 | 91.8 | 30.4 | 14.6 | 96.6 | 3.1 | 0.3 |  |
| 46 | Female | 22 | αα/αα | β*^HBB^*^:c.-23A>G^/β^N^ | 4.15 | 122 | 90.1 | 29.5 | 26.1 | 97.1 | 2.9 |  |  |
| 47 | Male | 23 | αα/αα | β*^HBB^*^:c.-23A>G^/β^N^ | 5.54 | 167 | 88.8 | 30.1 |  | 97.2 | 2.8 |  |  |
| 48 | Male | 23 | αα/αα | β*^HBB^*^:c.-23A>G^/β*^HBB^*^:c.126_129delCTTT^ | 6.32 | 130 | 62.8 | 20.6 |  | 94.6 | 5.4 |  |  |
| 49 | Male | 33 | αα/αα | β*^HBB^*^:c.-23A>G^/β^N^ | 5.31 | 159 | 89.2 | 29.8 |  | 97.0 | 3.0 |  |  |
| 50 | Male | 27 | αα/αα | β*^HBB^*^:c.-23A>G^/β^N^ | 4.17 | 134 | 94.3 | 32.0 | 18.5 | 97.2 | 2.8 |  |  |
| 51 | Male | 25 | αα/αα | β*^HBB^*^:c.-23A>G^/β^N^ | 4.56 | 133 | 88.9 | 29.2 | 21.2 | 97.3 | 2.7 |  |  |
| 52 | Female | 31 | αα/αα | β*^HBB^*^:c.-23A>G^/β^N^ | 4.19 | 131 | 87.3 | 31.2 | 30.5 | 97.4 | 2.6 |  |  |
| 53 | Female | 23 | αα/αα | β*^HBB^*^:c.-23A>G^/β^N^ | 4.63 | 142 | 89.2 | 30.7 | 34.6 | 96.9 | 3.1 |  |  |
| 54 | Male | 23 | αα/αα | β*^HBB^*^:c.-23A>G^/β^N^ | 5.69 | 158 | 84.2 | 27.8 | 10.8^a^ | 97.4 | 2.6 |  |  |
| 55 | Female | 23 | αα/αα | β*^HBB^*^:c.-23A>G^/β^N^ | 4.13 | 126 | 87.1 | 30.5 | 50.3 | 97.3 | 2.7 |  |  |
| 56 | Female | 20 | αα/αα | β*^HBB^*^:c.-23A>G^/β^N^ | 4.28 | 128 | 92.6 | 29.9 | 21.2 | 96.5 | 2.7 | 0.8 |  |
| 57 | Female | 20 | αα/αα | β*^HBB^*^:c.-23A>G^/β^N^ | 4.46 | 129 | 91.1 | 28.9 | 20 | 97.1 | 2.9 |  |  |
| 58 | Female | 24 | αα/αα | β*^HBB^*^:c.-23A>G^/β^N^ | 3.59 | 114 | 98.9 | 31.8 | 30.3 | 97.2 | 2.8 |  |  |
| 59 | Female | 22 | αα/αα | β*^HBB^*^:c.-23A>G^/β^N^ | 4.10 | 141 | 97.1 | 34.5 | 20.6 | 97.4 | 2.6 |  |  |
| 60^c^ | Male | 26 | αα/αα | β*^HBB^*^:c.-23A>G^/β^N^ | 5.57 | 176 | 87.9 | 31.6 | 22.9 | 97.1 | 2.9 |  | Wife's genotype: αα/αα β*^HBB^*^:c.126_129delCTTT^/β^N^. |
| 61 | Female | 28 | αα/αα | β*^HBB^*^:c.-23A>G^/β^N^ | 4.16 | 124 | 89.6 | 29.7 | 17.7 | 96.0 | 2.5 | 1.5 |  |
| 62 | Male | 25 | αα/-α^3.7^ | β*^HBB^*^:c.-23A>G^/β^N^ | 5.88 | 160 | 82.6 | 27.2 |  | 97.4 | 2.6 |  |  |
| 63 | Male | 26 | αα/αα | β*^HBB^*^:c.-23A>G^/β^N^ | 6.04 | 170 | 89.9 | 28.1 |  | 97.5 | 2.5 |  |  |
| 64 | Female | 22 | αα/αα | β*^HBB^*^:c.-23A>G^/β^N^ | 5.13 | 139 | 86.9 | 28.1 | 16.2 | 97.5 | 2.5 |  |  |
| 65 | Male | 25 | αα/αα | β*^HBB^*^:c.-23A>G^/β^N^ | 4.51 | 139 | 96.9 | 30.8 |  | 97.4 | 2.6 |  |  |
| 66 | Male | 27 | αα/αα | β*^HBB^*^:c.-23A>G^/β^N^ | 5.59 | 164 | 88.2 | 29.3 | 15.1 | 97.2 | 2.8 |  |  |
| 67 | Female | 28 | αα/--^SEA^ | β*^HBB^*^:c.-23A>G^/β^N^ | 5.75 | 121 | 66.8 | 21.0 | 22.9 | 97.8 | 2.2 |  |  |
| 68 | Male | 29 | αα/αα | β*^HBB^*^:c.-23A>G^/β^N^ | 4.95 | 155 | 88.9 | 31.3 |  | 97.6 | 2.4 |  |  |
| 69 | Male | 25 | αα/αα | β*^HBB^*^:c.-23A>G^/β^N^ | 5.81 | 175 | 89.2 | 30.1 | 36.1 | 97.3 | 2.7 |  |  |
| 70 | Female | 26 | αα/αα | β*^HBB^*^:c.-23A>G^/β^N^ | 3.65 | 121 | 99.9 | 33.3 | 40.8 | 97.3 | 2.7 |  |  |
| 71 | Male | 30 | αα/αα | β*^HBB^*^:c.-23A>G^/β^N^ | 4.99 | 147 | 88.6 | 29.4 | 28.7 | 97.4 | 2.6 |  |  |
| 72 | Male | 24 | αα/--^SEA^ | β*^HBB^*^:c.-23A>G^/β^N^ | 6.25 | 140 | 70.1 | 22.4 |  | 97.7 | 2.3 |  |  |
| 73 | Male | 32 | αα/αα | β*^HBB^*^:c.-23A>G^/β^N^ | 4.84 | 144 | 92.1 | 29.8 |  | 97.5 | 2.5 |  |  |
| 74 | Female | 25 | αα/αα | β*^HBB^*^:c.-23A>G^/β^N^ | 3.81 | 113 | 91.1 | 29.5 | 57.2 | 97.2 | 2.8 |  |  |
| 75 | Male | 27 | αα/αα | β*^HBB^*^:c.-23A>G^/β^N^ | 4.98 | 152 | 88.7 | 30.5 |  | 97.4 | 2.6 |  |  |
| ^a^Because the patient was diagnosed with iron-deficiency anemia, the data was excluded in the subsequent data analysis. ^b^The two cases were confirmed to be sisters during the follow-up. ^c^The spouses of these cases were found to carry β-thalassemia during follow-up. | | | | | | | | | | | | | |

**Table S3.** Clinical Information and Hematological Data of 20 Families with Carriers of the *HBB*: c.-23A>G Mutation Site.

| Case ID | Member | Sex | Age | α-globin Genotype | β-globin Genotype | RBC (10^12^/L) | HB (g/L) | MCV (fL) | MCH (pg) | Ferritin (ug/L) | Hb A (%) | Hb A2 (%) | Hb F (%) | Notes |
| --- | --- | --- | --- | --- | --- | --- | --- | --- | --- | --- | --- | --- | --- | --- |
| 21 | Proband | Female | 31 | αα/αα | β*^HBB^*^:c.-23A>G^/β*^HBB^*^:c.316-197C>T^ | 5.80 | 125 | 68.8 | 21.6 | 41.3 | 95.2 | 4.8 |  |  |
|  | Husband | Male | 30 | αα/αα | β^N^/β^N^ | 4.56 | 142 | 91.9 | 31.0 |  | 97.7 | 2.3 |  |  |
|  | Father | Male | 52 | αα/αα | β*^HBB^*^:c.-23A>G^/β^N^ | 5.19 | 159 | 93.9 | 30.7 |  | 97.4 | 2.6 |  |  |
|  | Mother | Female | 52 | αα/αα | β*^HBB^*^:c.316-197C>T^/β^N^ | 5.19 | 99 | 60.6 | 19.1 |  | 94.9 | 5.1 |  |  |
|  | Daughter | Female | 4 | αα/αα | β*^HBB^*^:c.-23A>G^/β^N^ | 4.44 | 128 | 86.8 | 28.8 |  | 97.3 | 2.7 |  |  |
| 48 | Proband | Male | 23 | αα/αα | β*^HBB^*^:c.-23A>G^/β*^HBB^*^:c.126_129delCTTT^ | 6.32 | 130 | 62.8 | 20.6 |  | 94.6 | 5.4 |  |  |
|  | Wife | Female | 23 | αα/αα | β^N^/β^N^ | 3.59 | 113 | 97.1 | 31.5 | 29.5 | 97.2 | 2.8 |  |  |
|  | Father | Male | 55 | αα/αα | β*^HBB^*^:c.126_129delCTTT^/β^N^ | 5.76 | 119 | 68.7 | 20.7 |  | 95.0 | 5.0 |  |  |
|  | Mother | Female | 48 | αα/αα | β*^HBB^*^:c.-23A>G^/β^N^ | 4.44 | 133 | 92.8 | 30.0 | 77.2 | 97.4 | 2.6 |  |  |
|  | Son | Male | 3 | αα/αα | β*^HBB^*^:c.126_129delCTTT^/β^N^ | 5.38 | 105 | 62.9 | 19.5 | 46.7 | 94.7 | 5.3 |  |  |
| 1 | Proband | Female | 26 | αα/αα | β*^HBB^*^:c.-23A>G^/β^N^ | 3.77 | 115 | 94.8 | 30.4 | 80.8 | 97.1 | 2.9 |  |  |
|  | Husband | Male | 28 | αα/αα | β^N^/β^N^ | 4.75 | 149 | 93.4 | 31.4 |  | 97.1 | 2.9 |  |  |
|  | Son | Male | 1 | αα/αα | β*^HBB^*^:c.-23A>G^/β^N^ | 4.15 | 107 | 82.0 | 25.7 | 4.02^a^ | 97.5 | 2.5 |  |  |
| 7 | Proband | Female | 24 | αα/αα | β*^HBB^*^:c.-23A>G^/β^N^ | 4.22 | 132 | 89.9 | 31.3 | 10.2^a^ | 97.5 | 2.5 |  |  |
|  | Husband | Male | 25 | αα/αα | β^N^/β^N^ | 5.52 | 157 | 87.6 | 28.4 |  | 97.5 | 2.5 |  |  |
|  | Son | Male | 6M | αα/αα | β^N^/β^N^ | 4.33 | 117 | 83.3 | 26.7 | 68.6 | 93.8 | 2.7 | 3.5 |  |
| 8 | Proband | Female | 29 | αα/αα | β*^HBB^*^:c.-23A>G^/β^N^ | 3.89 | 124 | 91.2 | 31.8 | 37.7 | 96.0 | 2.8 | 1.2 |  |
|  | Husband | Male | 29 | αα/αα | β^N^/β^N^ | 5.25 | 165 | 93.3 | 31.4 |  | 96.9 | 2.7 | 0.4 |  |
|  | Daughter | Female | 4 | αα/αα | β^N^/β^N^ | 4.86 | 141 | 89.6 | 29.0 | 56.7 | 96.9 | 2.8 | 0.3 |  |
| 9^b^ | Proband | Male | 33 | αα/αα | β*^HBB^*^:c.-23A>G^/β^N^ | 5.30 | 157 | 97.9 | 29.6 |  | 97.4 | 2.6 |  | Prenatal diagnosis was conducted in April 2020, and the fetal genotype was:αα/αα β*^HBB^*^:c.316-197C>T^/β^N^. |
|  | Wife | Female | 32 | αα/αα | β*^HBB^*^:c.316-197C>T^/β^N^ | 5.07 | 99 | 63.9 | 19.5 | 142 | 94.2 | 4.9 | 0.9 |  |
|  | Son | Male | 4 | αα/αα | β*^HBB^*^:c.316-197C>T^/β^N^ | 6.13 | 104 | 56.5 | 17.0 | 23.6 | 94.4 | 5.3 | 0.3 |  |
| 11 | Proband | Female | 27 | αα/αα | β*^HBB^*^:c.-23A>G^/β^N^ | 4.01 | 126 | 99.8 | 31.5 | 46.3 | 97.3 | 2.7 |  |  |
|  | Husband | Male | 25 | αα/αα | β^N^/β^N^ | 5.09 | 143 | 88.9 | 28.1 |  | 97.1 | 2.9 |  |  |
|  | Son | Male | 1 | αα/αα | β*^HBB^*^:c.-23A>G^/β^N^ | 5.48 | 138 | 77.8 | 25.2 | 8.9^a^ | 93.9 | 2.7 | 3.4 |  |
| 12 | Proband | Female | 40 | αα/αα | β*^HBB^*^:c.-23A>G^/β^N^ | 4.27 | 127 | 87.3 | 29.8 |  | 97.2 | 2.8 |  |  |
|  | Husband | Male | 37 | αα/αα | β^N^/β^N^ | 5.27 | 164 | 93.5 | 31.1 |  | 97.2 | 2.8 |  |  |
|  | Son | Male | 6 | αα/αα | β^N^/β^N^ | 4.57 | 127 | 84.1 | 27.7 |  | 96.9 | 2.7 | 0.4 |  |
| 13 | Proband | Female | 31 | αα/--^SEA^ | β*^HBB^*^:c.-23A>G^/β^N^ | 5.24 | 114 | 73.4 | 21.8 | 28.2 | 97.7 | 2.3 |  |  |
|  | Husband | Male | 29 | αα/αα | β^N^/β^N^ | 3.95 | 131 | 102.5 | 33.3 | 44.3 | 97.4 | 2.6 |  |  |
|  | Daughter | Female | 3 | αα/αα | β*^HBB^*^:c.-23A>G^/β^N^ | 4.45 | 131 | 89.2 | 29.4 | 79.2 | 97.3 | 2.7 |  |  |
| 15 | Proband | Male | 36 | αα/--^SEA^ | β*^HBB^*^:c.-23A>G^/β^N^ | 5.36 | 110 | 69.5 | 20.4 |  | 97.8 | 2.2 |  |  |
|  | Wife | Female | 33 | αα/αα | β^N^/β^N^ | 4.45 | 137 | 92.3 | 30.8 | 89.2 | 97.4 | 2.6 |  |  |
|  | Daughter | Female | 3 | αα/--^SEA^ | β^N^/β^N^ | 5.84 | 120 | 68.1 | 20.5 | 67.3 | 97.2 | 2.5 | 0.3 |  |
| 18 | Proband | Female | 26 | αα/αα | β*^HBB^*^:c.-23A>G^/β^N^ | 4.38 | 141 | 92.9 | 32.3 | 30.5 | 96.7 | 2.7 | 0.6 |  |
|  | Husband | Male | 27 | αα/αα | β^N^/β^N^ | 5.58 | 162 | 91.0 | 29.0 |  | 97.6 | 2.4 |  |  |
|  | Son | Male | 7M | αα/αα | β^N^/β^N^ | 4.90 | 87 | 64.0 | 17.7 | 3.03^a^ | 97.4 | 1.8 | 0.8 |  |
| 27 | Proband | Female | 28 | αα/αα | β*^HBB^*^:c.-23A>G^/β^N^ | 4.17 | 130 | 93.4 | 31.1 | 25.3 | 97.2 | 2.8 |  |  |
|  | Husband | Male | 34 | αα/αα | β^N^/β^N^ | 5.43 | 163 | 92.2 | 30.0 |  | 97.5 | 2.5 |  |  |
|  | Daughter | Female | 8 | αα/αα | β*^HBB^*^:c.-23A>G^/β^N^ | 4.18 | 120 | 87.8 | 28.8 | 30.3 | 97.3 | 2.7 |  |  |
| 35 | Proband | Female | 20 | αα/αα | β*^HBB^*^:c.-23A>G^/β^N^ | 4.49 | 103 | 72.0 | 22.9 | 6.9^a^ | 97.7 | 2.3 |  |  |
|  | Husband | Male | 22 | αα/αα | β^N^/β^N^ | 5.64 | 161 | 88.4 | 28.5 |  | 97.4 | 2.6 |  |  |
|  | Daughter | Female | 1 | αα/αα | β*^HBB^*^:c.-23A>G^/β^N^ | 4.47 | 127 | 85.7 | 28.5 | 16.9 | 96.4 | 2.8 | 0.8 |  |
| 46 | Proband | Female | 26 | αα/αα | β^N^/β^N^ | 4.15 | 122 | 90.1 | 29.5 | 26.1 | 97.1 | 2.9 |  |  |
|  | Husband | Male | 26 | αα/αα | β^N^/β^N^ | 4.91 | 151 | 95.3 | 30.6 |  | 97.4 | 2.6 |  |  |
|  | Daughter | Female | 3 | αα/αα | β^N^/β^N^ | 4.23 | 120 | 88.2 | 28.5 | 38.7 | 97.3 | 2.7 |  |  |
|  | Daughter | Female | 1 | αα/αα | β^N^/β^N^ | 5.15 | 121 | 74.6 | 23.5 | 4.6^a^ | 95.3 | 2.2 | 2.5 |  |
| 49 | Proband | Male | 33 | αα/αα | β*^HBB^*^:c.-23A>G^/β^N^ | 5.31 | 159 | 89.2 | 29.8 |  | 97.0 | 3.0 |  |  |
|  | Wife | Female | 33 | αα/αα | β^N^/β^N^ | 4.36 | 132 | 93.3 | 30.4 | 43.9 | 96.5 | 2.9 | 0.6 |  |
|  | Son | Male | 8 | αα/αα | β*^HBB^*^:c.-23A>G^/β^N^ | 3.59 | 117 | 98.0 | 32.5 | 38.7 | 97.2 | 2.8 |  |  |
| 57 | Proband | Female | 25 | αα/αα | β*^HBB^*^:c.-23A>G^/β^N^ | 4.46 | 129 | 91.1 | 28.9 | 20 | 97.1 | 2.9 |  |  |
|  | Husband | Male | 26 | αα/αα | β^N^/β^N^ | 4.96 | 149 | 89.4 | 30.0 |  | 97.0 | 3.0 |  |  |
|  | Daughter | Female | 4 | αα/αα | β^N^/β^N^ | 4.75 | 138 | 90.2 | 29.2 | 29.4 | 97.4 | 2.6 |  |  |
|  | Son | Male | 2 | αα/αα | β*^HBB^*^:c.-23A>G^/β^N^ | 4.47 | 124 | 81.2 | 27.7 | 11.6^a^ | 97.0 | 3.0 |  |  |
| 60 | Proband | Male | 26 | αα/αα | β*^HBB^*^:c.-23A>G^/β^N^ | 5.57 | 176 | 87.9 | 31.6 | 22.9 | 97.1 | 2.9 |  |  |
|  | Wife | Female | 26 | αα/αα | β*^HBB^*^:c.126_129delCTTT^/ β^N^ | 5.36 | 112 | 62.4 | 20.9 |  | 93.6 | 5.7 | 0.7 |  |
|  | Son | Male | 11M | αα/αα | β^N^/β^N^ | 4.49 | 129 | 84.2 | 28.7 | 15.7 | 94.5 | 2.9 | 2.6 |  |
| 62 | Proband | Male | 25 | αα/-α^3.7^ | β^N^/β^N^ | 5.88 | 160 | 82.6 | 27.2 |  | 97.4 | 2.6 |  |  |
|  | Wife | Female | 26 | αα/αα | β^N^/β^N^ | 3.76 | 111 | 86.9 | 29.5 | 30 | 97.7 | 2.3 |  |  |
|  | Daughter | Female | 6M | αα/αα | β^N^/β^N^ | 4.70 | 120 | 84.8 | 26.9 | 75.8 | 94.0 | 2.0 | 4.0 |  |
| 70 | Proband | Female | 31 | αα/αα | β*^HBB^*^:c.-23A>G^/β^N^ | 3.65 | 121 | 99.9 | 33.3 | 40.8 | 97.3 | 2.7 |  |  |
|  | Husband | Male | 32 | αα/αα | β^N^/β^N^ | 4.84 | 158 | 95.7 | 32.7 |  | 96.7 | 2.8 | 0.5 |  |
|  | Daughter | Female | 4 | αα/αα | β*^HBB^*^:c.-23A>G^/β^N^ | 3.53 | 109 | 91.5 | 30.7 | 35.3 | 97.5 | 2.5 |  |  |
|  | Daughter | Female | 1 | αα/αα | β^N^/β^N^ | 4.38 | 123 | 83.9 | 28.2 | 28.2 | 95.8 | 3.0 | 1.2 |  |
| 74 | Proband | Female | 25 | αα/αα | β*^HBB^*^:c.-23A>G^/β^N^ | 3.81 | 113 | 91.1 | 29.5 | 57.2 | 97.2 | 2.8 |  |  |
|  | Husband | Male | 26 | αα/--^SEA^ | β^N^/β^N^ | 6.47 | 141 | 70.5 | 21.9 |  | 97.6 | 2.4 |  |  |
|  | Son | Male | 1 | αα/--^SEA^ | β^N^/β^N^ | 6.05 | 115 | 63.4 | 19.0 | 6.8^a^ | 97.3 | 2.3 | 0.4 |  |
| ^a^Because the patient was diagnosed with iron-deficiency anemia, the data was excluded in the subsequent data analysis. ^b^Prenatal diagnosis was performed in this case because both spouses carried mutations in the β-globin gene. | | | | | | | | | | | | | | |

**Table S4.** Hematological Data of *HBB*: c.-23A>G Mutation Carriers (75 Carriers and Their Family Members Confirmed to Carry the Mutation) After Excluding Individuals with Iron-Deficiency Anemia.

| Case ID | Sex | Age | α-globin Genotype | β-globin Genotype | RBC (10^12^/L) | HB (g/L) | MCV (fL) | MCH (pg) | Ferritin (ug/L) | Hb A (%) | Hb A2 (%) | Hb F (%) | Notes |
| --- | --- | --- | --- | --- | --- | --- | --- | --- | --- | --- | --- | --- | --- |
| 1 | Female | 26 | αα/αα | β*^HBB^*^:c.-23A>G^/β^N^ | 3.77 | 115 | 94.8 | 30.4 | 80.8 | 97.1 | 2.9 |  |  |
| 2 | Female | 23 | αα/αα | β*^HBB^*^:c.-23A>G^/β^N^ | 3.46 | 111 | 98.6 | 32.2 | 50.5 | 97.1 | 2.9 |  |  |
| 3 | Female | 33 | αα/αα | β*^HBB^*^:c.-23A>G^/β^N^ | 3.70 | 118 | 96.7 | 31.8 | 33.9 | 97.4 | 2.6 |  |  |
| 4 | Male | 40 | αα/αα | β*^HBB^*^:c.-23A>G^/β^N^ | 5.33 | 167 | 92.9 | 31.3 |  | 97.6 | 2.4 |  |  |
| 5 | Female | 28 | αα/αα | β*^HBB^*^:c.-23A>G^/β^N^ | 3.76 | 119 | 96.9 | 31.7 | 16.9 | 97.3 | 2.3 | 0.4 |  |
| 6 | Female | 32 | αα/αα | β*^HBB^*^:c.-23A>G^/β^N^ | 3.19 | 111 | 99.5 | 34.8 | 33.9 | 97.3 | 2.7 |  |  |
| 8 | Female | 29 | αα/αα | β*^HBB^*^:c.-23A>G^/β^N^ | 3.89 | 124 | 91.2 | 31.8 | 37.7 | 96.0 | 2.8 | 1.2 |  |
| 9 | Male | 33 | αα/αα | β*^HBB^*^:c.-23A>G^/β^N^ | 5.30 | 157 | 97.9 | 29.6 |  | 97.4 | 2.6 |  |  |
| 11 | Female | 27 | αα/αα | β*^HBB^*^:c.-23A>G^/β^N^ | 4.01 | 126 | 99.8 | 31.5 | 46.3 | 97.3 | 2.7 |  |  |
| 12 | Female | 40 | αα/αα | β*^HBB^*^:c.-23A>G^/β^N^ | 4.27 | 127 | 87.3 | 29.8 |  | 97.2 | 2.8 |  |  |
| 14 | Female | 28 | αα/αα | β*^HBB^*^:c.-23A>G^/β^N^ | 4.77 | 148 | 96.3 | 31.0 | 55.1 | 97.6 | 2.4 |  |  |
| 16 | Female | 30 | αα/αα | β*^HBB^*^:c.-23A>G^/β^N^ | 4.19 | 115 | 84.8 | 27.5 | 40.4 | 97.4 | 2.6 |  |  |
| 17 | Female | 23 | αα/αα | β*^HBB^*^:c.-23A>G^/β^N^ | 5.08 | 147 | 88.9 | 29.0 | 70.4 | 97.3 | 2.7 |  |  |
| 18 | Female | 26 | αα/αα | β*^HBB^*^:c.-23A>G^/β^N^ | 4.38 | 141 | 92.9 | 32.3 | 30.5 | 96.7 | 2.7 | 0.6 |  |
| 19 | Male | 27 | αα/αα | β*^HBB^*^:c.-23A>G^/β^N^ | 5.10 | 159 | 93.2 | 31.2 |  | 97.5 | 2.5 |  |  |
| 20 | Female | 30 | αα/αα | β*^HBB^*^:c.-23A>G^/β^N^ | 4.23 | 123 | 89.7 | 29.0 | 39.2 | 97.1 | 2.9 |  |  |
| 22 | Female | 25 | αα/αα | β*^HBB^*^:c.-23A>G^/β^N^ | 3.59 | 111 | 93.8 | 30.8 | 37 | 97.4 | 2.6 |  |  |
| 24 | Female | 23 | αα/αα | β*^HBB^*^:c.-23A>G^/β^N^ | 4.23 | 128 | 90.3 | 30.3 | 27.3 | 97.3 | 2.7 |  |  |
| 25 | Female | 25 | αα/αα | β*^HBB^*^:c.-23A>G^/β^N^ | 4.20 | 130 | 93.0 | 31.0 | 17.6 | 97.4 | 2.6 |  |  |
| 26 | Female | 33 | αα/αα | β*^HBB^*^:c.-23A>G^/β^N^ | 3.59 | 111 | 96.1 | 31.0 | 15.5 | 97.3 | 2.7 |  |  |
| 27 | Female | 28 | αα/αα | β*^HBB^*^:c.-23A>G^/β^N^ | 4.17 | 130 | 93.4 | 31.1 | 25.3 | 97.2 | 2.8 |  |  |
| 29 | Female | 22 | αα/αα | β*^HBB^*^:c.-23A>G^/β^N^ | 4.25 | 130 | 92.4 | 30.6 | 46.2 | 97.5 | 2.5 |  |  |
| 30 | Female | 31 | αα/αα | β*^HBB^*^:c.-23A>G^/β^N^ | 4.02 | 125 | 94.1 | 31.1 | 37.9 | 97.4 | 2.6 |  |  |
| 32 | Female | 20 | αα/αα | β*^HBB^*^:c.-23A>G^/β^N^ | 3.83 | 116 | 90.8 | 30.2 | 16.6 | 97.4 | 2.6 |  |  |
| 33 | Female | 24 | αα/αα | β*^HBB^*^:c.-23A>G^/β^N^ | 3.80 | 118 | 93.9 | 31.0 | 112 | 97.0 | 3.0 |  |  |
| 34 | Female | 26 | αα/αα | β*^HBB^*^:c.-23A>G^/β^N^ | 4.27 | 129 | 94.3 | 30.3 | 23 | 97.0 | 3.0 |  |  |
| 36 | Female | 20 | αα/αα | β*^HBB^*^:c.-23A>G^/β^N^ | 4.10 | 144 | 97.8 | 35.1 | 14.7 | 97.3 | 2.7 |  |  |
| 37 | Male | 25 | αα/αα | β*^HBB^*^:c.-23A>G^/β^N^ | 4.96 | 153 | 88.3 | 30.9 |  | 97.4 | 2.6 |  |  |
| 38 | Female | 20 | αα/αα | β*^HBB^*^:c.-23A>G^/β^N^ | 3.59 | 118 | 99.1 | 32.7 | 35.3 | 97.1 | 2.9 |  |  |
| 39 | Male | 23 | αα/αα | β*^HBB^*^:c.-23A>G^/β^N^ | 5.47 | 162 | 85.3 | 29.7 |  | 97.2 | 2.8 |  |  |
| 40 | Male | 23 | αα/αα | β*^HBB^*^:c.-23A>G^/β^N^ | 4.82 | 151 | 95.4 | 31.3 | 68.2 | 97.0 | 3.0 |  |  |
| 41 | Male | 24 | αα/αα | β*^HBB^*^:c.-23A>G^/β^N^ | 5.45 | 159 | 89.1 | 29.1 | 18.6 | 97.5 | 2.5 |  |  |
| 42 | Male | 36 | αα/αα | β*^HBB^*^:c.-23A>G^/β^N^ | 5.41 | 154 | 88.8 | 28.5 |  | 97.4 | 2.6 |  |  |
| 43 | Male | 35 | αα/αα | β*^HBB^*^:c.-23A>G^/β^N^ | 4.56 | 132 | 91.4 | 28.9 | 27.2 | 97.4 | 2.6 |  |  |
| 44 | Male | 26 | αα/αα | β*^HBB^*^:c.-23A>G^/β^N^ | 4.73 | 151 | 94.4 | 31.9 | 53.2 | 97.5 | 2.5 |  |  |
| 45 | Female | 23 | αα/αα | β*^HBB^*^:c.-23A>G^/β^N^ | 3.98 | 121 | 91.8 | 30.4 | 14.6 | 96.6 | 3.1 | 0.3 |  |
| 46 | Female | 22 | αα/αα | β*^HBB^*^:c.-23A>G^/β^N^ | 4.15 | 122 | 90.1 | 29.5 | 26.1 | 97.1 | 2.9 |  |  |
| 47 | Male | 23 | αα/αα | β*^HBB^*^:c.-23A>G^/β^N^ | 5.54 | 167 | 88.8 | 30.1 |  | 97.2 | 2.8 |  |  |
| 49 | Male | 33 | αα/αα | β*^HBB^*^:c.-23A>G^/β^N^ | 5.31 | 159 | 89.2 | 29.8 |  | 97.0 | 3.0 |  |  |
| 50 | Male | 27 | αα/αα | β*^HBB^*^:c.-23A>G^/β^N^ | 4.17 | 134 | 94.3 | 32.0 | 18.5 | 97.2 | 2.8 |  |  |
| 51 | Male | 25 | αα/αα | β*^HBB^*^:c.-23A>G^/β^N^ | 4.56 | 133 | 88.9 | 29.2 | 21.2 | 97.3 | 2.7 |  |  |
| 52 | Female | 31 | αα/αα | β*^HBB^*^:c.-23A>G^/β^N^ | 4.19 | 131 | 87.3 | 31.2 | 30.5 | 97.4 | 2.6 |  |  |
| 53 | Female | 23 | αα/αα | β*^HBB^*^:c.-23A>G^/β^N^ | 4.63 | 142 | 89.2 | 30.7 | 34.6 | 96.9 | 3.1 |  |  |
| 55 | Female | 23 | αα/αα | β*^HBB^*^:c.-23A>G^/β^N^ | 4.13 | 126 | 87.1 | 30.5 | 50.3 | 97.3 | 2.7 |  |  |
| 56 | Female | 20 | αα/αα | β*^HBB^*^:c.-23A>G^/β^N^ | 4.28 | 128 | 92.6 | 29.9 | 21.2 | 96.5 | 2.7 | 0.8 |  |
| 57 | Female | 20 | αα/αα | β*^HBB^*^:c.-23A>G^/β^N^ | 4.46 | 129 | 91.1 | 28.9 | 20 | 97.1 | 2.9 |  |  |
| 58 | Female | 24 | αα/αα | β*^HBB^*^:c.-23A>G^/β^N^ | 3.59 | 114 | 98.9 | 31.8 | 30.3 | 97.2 | 2.8 |  |  |
| 59 | Female | 22 | αα/αα | β*^HBB^*^:c.-23A>G^/β^N^ | 4.10 | 141 | 97.1 | 34.5 | 20.6 | 97.4 | 2.6 |  |  |
| 60 | Male | 26 | αα/αα | β*^HBB^*^:c.-23A>G^/β^N^ | 5.57 | 176 | 87.9 | 31.6 | 22.9 | 97.1 | 2.9 |  |  |
| 61 | Female | 28 | αα/αα | β*^HBB^*^:c.-23A>G^/β^N^ | 4.16 | 124 | 89.6 | 29.7 | 17.7 | 96.0 | 2.5 | 1.5 |  |
| 63 | Male | 26 | αα/αα | β*^HBB^*^:c.-23A>G^/β^N^ | 6.04 | 170 | 89.9 | 28.1 |  | 97.5 | 2.5 |  |  |
| 64 | Female | 22 | αα/αα | β*^HBB^*^:c.-23A>G^/β^N^ | 5.13 | 139 | 86.9 | 28.1 | 16.2 | 97.5 | 2.5 |  |  |
| 65 | Male | 25 | αα/αα | β*^HBB^*^:c.-23A>G^/β^N^ | 4.51 | 139 | 96.9 | 30.8 |  | 97.4 | 2.6 |  |  |
| 66 | Male | 27 | αα/αα | β*^HBB^*^:c.-23A>G^/β^N^ | 5.59 | 164 | 88.2 | 29.3 | 15.1 | 97.2 | 2.8 |  |  |
| 68 | Male | 29 | αα/αα | β*^HBB^*^:c.-23A>G^/β^N^ | 4.95 | 155 | 88.9 | 31.3 |  | 97.6 | 2.4 |  |  |
| 69 | Male | 25 | αα/αα | β*^HBB^*^:c.-23A>G^/β^N^ | 5.81 | 175 | 89.2 | 30.1 | 36.1 | 97.3 | 2.7 |  |  |
| 70 | Female | 26 | αα/αα | β*^HBB^*^:c.-23A>G^/β^N^ | 3.65 | 121 | 99.9 | 33.3 | 40.8 | 97.3 | 2.7 |  |  |
| 71 | Male | 30 | αα/αα | β*^HBB^*^:c.-23A>G^/β^N^ | 4.99 | 147 | 88.6 | 29.4 | 28.7 | 97.4 | 2.6 |  |  |
| 73 | Male | 32 | αα/αα | β*^HBB^*^:c.-23A>G^/β^N^ | 4.84 | 144 | 92.1 | 29.8 |  | 97.5 | 2.5 |  |  |
| 74 | Female | 25 | αα/αα | β*^HBB^*^:c.-23A>G^/β^N^ | 3.81 | 113 | 91.1 | 29.5 | 57.2 | 97.2 | 2.8 |  |  |
| 75 | Male | 27 | αα/αα | β*^HBB^*^:c.-23A>G^/β^N^ | 4.98 | 152 | 88.7 | 30.5 |  | 97.4 | 2.6 |  |  |
| 21* | Male | 52 | αα/αα | β*^HBB^*^:c.-23A>G^/β^N^ | 5.19 | 159 | 93.9 | 30.7 |  | 97.4 | 2.6 |  | Father |
| 21* | Female | 4 | αα/αα | β*^HBB^*^:c.-23A>G^/β^N^ | 4.44 | 128 | 86.8 | 28.8 |  | 97.3 | 2.7 |  | Daughter |
| 48* | Female | 48 | αα/αα | β*^HBB^*^:c.-23A>G^/β^N^ | 4.44 | 133 | 92.8 | 30.0 | 77.2 | 97.4 | 2.6 |  | Mother |
| 13* | Female | 3 | αα/αα | β*^HBB^*^:c.-23A>G^/β^N^ | 4.45 | 131 | 89.2 | 29.4 | 79.2 | 97.3 | 2.7 |  | Daughter |
| 27* | Female | 8 | αα/αα | β*^HBB^*^:c.-23A>G^/β^N^ | 4.18 | 120 | 87.8 | 28.8 | 30.3 | 97.3 | 2.7 |  | Daughter |
| 35* | Female | 1 | αα/αα | β*^HBB^*^:c.-23A>G^/β^N^ | 4.47 | 127 | 85.7 | 28.5 | 16.9 | 96.4 | 2.8 | 0.8 | Daughter |
| 49* | Male | 8 | αα/αα | β*^HBB^*^:c.-23A>G^/β^N^ | 3.59 | 117 | 98.0 | 32.5 | 38.7 | 97.2 | 2.8 |  | Son |
| 70* | Female | 4 | αα/αα | β*^HBB^*^:c.-23A>G^/β^N^ | 3.53 | 109 | 91.5 | 30.7 | 35.3 | 97.5 | 2.5 |  | Daughter |
| 10 | Female | 25 | αα/--^SEA^ | β*^HBB^*^:c.-23A>G^/β^N^ | 5.51 | 121 | 71.2 | 21.9 | 64.9 | 97.7 | 2.3 |  |  |
| 13 | Female | 31 | αα/--^SEA^ | β*^HBB^*^:c.-23A>G^/β^N^ | 5.24 | 114 | 73.4 | 21.8 | 28.2 | 97.7 | 2.3 |  |  |
| 15 | Male | 36 | αα/--^SEA^ | β*^HBB^*^:c.-23A>G^/β^N^ | 5.36 | 110 | 69.5 | 20.4 |  | 97.8 | 2.2 |  |  |
| 31 | Female | 38 | αα/--^SEA^ | β*^HBB^*^:c.-23A>G^/β^N^ | 5.48 | 120 | 69.4 | 22.0 | 56.7 | 97.6 | 2.4 |  |  |
| 67 | Female | 28 | αα/--^SEA^ | β*^HBB^*^:c.-23A>G^/β^N^ | 5.75 | 121 | 66.8 | 21.0 | 22.9 | 97.8 | 2.2 |  |  |
| 72 | Male | 24 | αα/--^SEA^ | β*^HBB^*^:c.-23A>G^/β^N^ | 6.25 | 140 | 70.1 | 22.4 |  | 97.7 | 2.3 |  |  |
| 62 | Male | 25 | αα/-α^3.7^ | β*^HBB^*^:c.-23A>G^/β^N^ | 5.88 | 160 | 82.6 | 27.2 |  | 97.4 | 2.6 |  |  |
| 21 | Female | 31 | αα/αα | β*^HBB^*^:c.-23A>G^/β*^HBB^*^:c.316-197C>T^ | 5.80 | 125 | 68.8 | 21.6 | 41.3 | 95.2 | 4.8 |  |  |
| 48 | Male | 23 | αα/αα | β*^HBB^*^:c.-23A>G^/β*^HBB^*^:c.126_129delCTTT^ | 6.32 | 130 | 62.8 | 20.6 |  | 94.6 | 5.4 |  |  |
| *These data were obtained through pedigree verification. | | | | | | | | | | | | | |
